# Secondary contact erodes Pleistocene diversification in a wide‐ranging freshwater mussel *(Quadrula*)

**DOI:** 10.1111/mec.17572

**Published:** 2024-11-14

**Authors:** Sean M. Keogh, Nathan A. Johnson, Chase H. Smith, Bernard E. Sietman, Jeffrey T. Garner, Charles R. Randklev, Andrew M. Simons

**Affiliations:** ^1^ Gantz Family Collections Center, Field Museum of Natural History Chicago Illinois USA; ^2^ Bell Museum of Natural History, University of Minnesota St. Paul Minnesota USA; ^3^ U.S. Geological Survey, Wetland and Aquatic Research Center Gainesville Florida USA; ^4^ Department of Integrative Biology University of Texas Austin Texas USA; ^5^ Minnesota Department of Natural Resources Center for Aquatic Mollusk Programs Lake City Minnesota USA; ^6^ Alabama Division of Wildlife and Freshwater Fisheries Florence Alabama USA; ^7^ Texas A&M Natural Resources Institute, AgriLife Research Center Dallas Texas USA; ^8^ Department of Fisheries, Wildlife, and Conservation Biology University of Minnesota St. Paul Minnesota USA

**Keywords:** freshwater mussels, introgression, lineage fusion, mitonuclear discordance, Pleistocene, secondary contact, species delimitation

## Abstract

The isolated river drainages of eastern North America serve as a natural laboratory to investigate the roles of allopatry and secondary contact in the evolutionary trajectories of recently diverged lineages. Drainage divides facilitate allopatric speciation, but due to their sensitivity to climatic and geomorphological changes, neighboring rivers frequently coalesce, creating recurrent opportunities of isolation and contact throughout the history of aquatic lineages. The freshwater mussel *Quadrula quadrula* is widely distributed across isolated rivers of eastern North America and possesses high phenotypic and molecular variation across its range. We integrate sequence data from three genomes, including female‐ and male‐inherited mitochondrial markers and thousands of nuclear encoded SNPs with morphology and geography to illuminate the group's divergence history. Across contemporary isolated rivers, we found continuums of molecular and morphological variation, following a pattern of isolation by distance. In contact zones, hybridization was frequent with no apparent fitness consequences, as advanced hybrids were common. Accordingly, we recognize *Q. quadrula* as a single cohesive species with subspecific variation (*Q. quadrula rumphiana*). Demographic modeling and divergence dating supported a divergence history characterized by allopatric vicariance followed by secondary contact, likely driven by river rearrangements and Pleistocene glacial cycles. Despite clinal range‐wide variation and hybridization in contact zones, the process‐based species delimitation tool *delimitR*, which considers demographic scenarios like secondary contact, supported the delimitation of the maximum number of species tested. As such, when interpreting species delimitation results, we suggest careful consideration of spatial sampling and subsequent geographic patterns of biological variation, particularly for wide‐ranging taxa.

## 1 Introduction

Differentiating within‐species population structure from independently evolving species is challenging (Carstens et al. 2013; Sukumaran and Knowles 2017). Yet the accurate diagnosis of species boundaries is critical for nearly all fields of biology, particularly biodiversity conservation, as species are frequently the unit of legislative protection (Coates, Byrne, and Moritz 2018; Mace 2004). Taxonomic decisions often rely solely on mitochondrial markers (mtDNA), but reliance on these markers can lead to incorrect decisions when mtDNA genealogies do not reflect the species tree (Edwards and Bensch 2009). Discordance of gene and species trees is a well understood phenomenon, as processes like gene flow, known to be common among closely related species, and incomplete lineage sorting can produce complex divergence histories (Funk and Omland 2003; McGuire et al. 2007). However, the improvement of DNA sequencing technologies and sophisticated species delimitation tools allows for the diagnosis of species boundaries in the face of these complexities. Additionally, these analyses can be combined with phenotypic and geographic data to holistically delimit independently evolving lineages (de Queiroz 2007).

Although there is disagreement among the levels of independence necessary for speciation to have taken place, we assert that the degree to which bifurcating populations are reproductively isolated determines the progress of speciation (Barth et al. 2020; Kearns et al. 2018; Slager et al. 2020). A direct test of reproductive isolation can be made where diverging lineages interact in sympatry, creating contact or hybrid zones (Mayr 1996). These zones provide a natural laboratory for investigating the maintenance of reproductive isolation, as contact can reinforce or erode differentiation between sympatric lineages (Johannesson et al. 2020). When reproductive isolation is incomplete, indiscriminate gene flow may disintegrate previously divergent lineages, resulting in a single, genetically admixed species. Alternatively, gene flow in contact zones can be rare if selection against hybrids results in assortative mating and the reinforcement of species boundaries. Populations that experience waves of recurrent allopatric isolation followed by secondary contact are ideal systems to understand the role of demography in speciation dynamics.

The isolated river drainages of eastern North America experienced repeated glacial expansion and recession throughout the Pleistocene (2.58 Ma—11.7 ka), resulting in cyclical periods of isolation and contact for riverine organisms (Soltis et al. 2006; Strange and Burr 1997). During glacial recession, southern river drainages were inundated by rising sea levels, eliminating southward freshwater connections between neighboring river basins. During glacial expansion, sea‐level recession expanded river length southward into the Gulf of Mexico, forging connections among previously isolated river basins (Anderson et al. 2004; Fildani et al. 2018; Hewitt 2004; Swift et al. 1986). Phylogeography and divergence dating of many North American freshwater taxa show congruence between the timing of Pleistocene climatic fluctuations and diversification events, suggesting drainage capture and isolation have played a dramatic role in the proliferation of aquatic diversity (April et al. 2013; Inoue et al. 2014; Johnson et al. 2023; Keogh et al. 2021; Near and Benard 2004).

**FIGURE 1:**
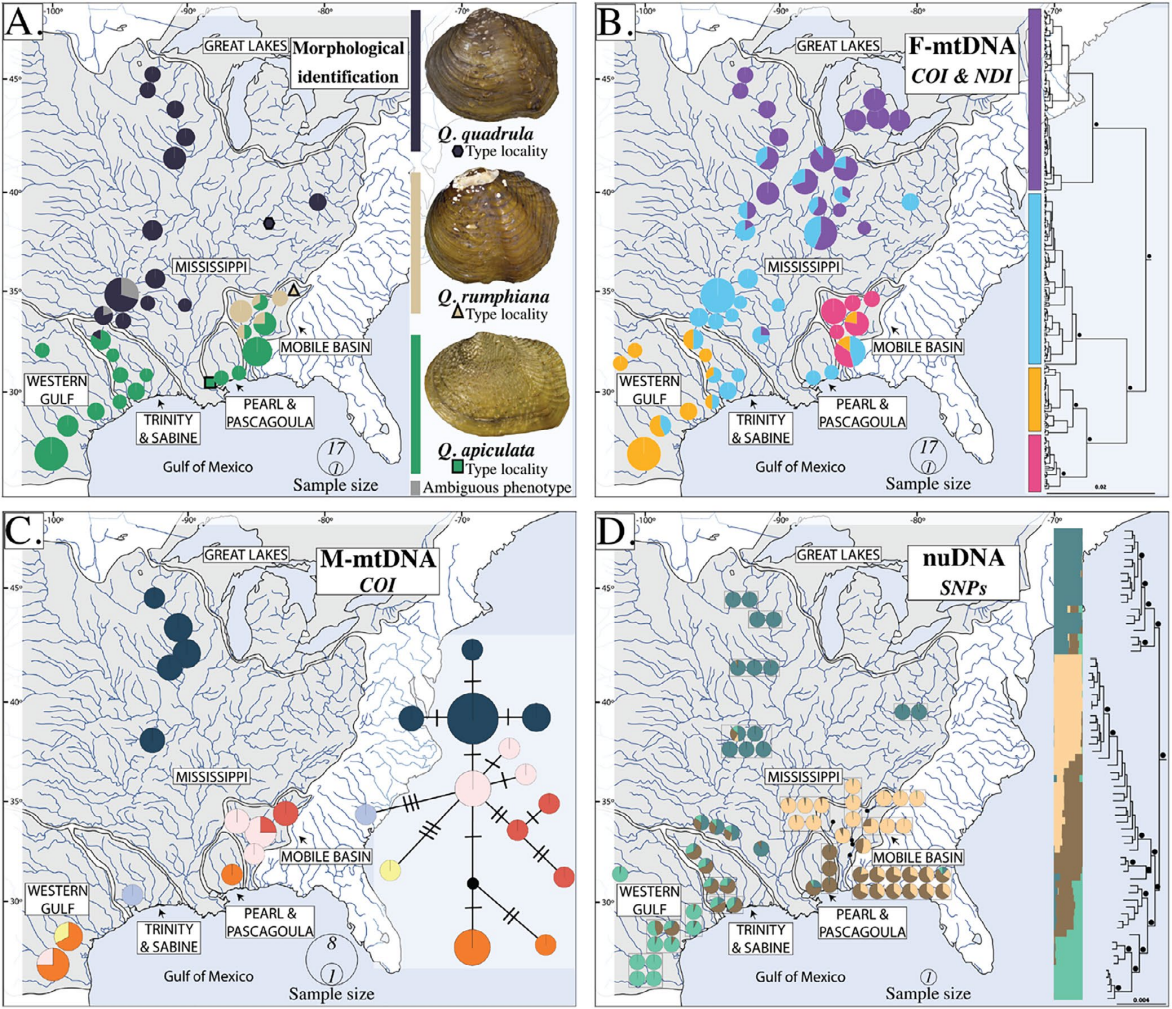
Map of eastern North America and the spatial distribution of (A) sampled shells identified to putative species: *Q. quadrula*, *Q. rumphiana*, and *Q. apiculata*. Photos of shells: Neotype of *Obliquaria (Quadrula) quadrula* Rafinesque 1820 (ANSP 20224), holotype of *Unio rumphianus* Lea 1852 (USNM 84189), and syntype of *Unio speciosus* Lea  1862 (junior synonym of *Q. apiculata*) (USNM 84172). Photos provided by the National Museum of Natural History and P. Callomon at the Academy of the Natural Sciences of Drexel University. (B) Mapping of female‐inherited mitochondrial clades (F‐mtDNA: COI & NDI) across sampled localities and their estimated phylogenetic relationships recovered from BEAST v2.6.6. Black dots are placed at informative nodes that indicate > 0.85 posterior probability. (C) Spatial distribution of male‐inherited (M‐mtDNA: COI) haplotypes and TCS haplotype network. In A–C, sampled localities were combined when they occurred within 100 km of a contiguous river system from each other. (D) sNMF ancestry coefficients for each sampled individual. Phylogeny produced from the ‘msl17’ dataset in IQ‐TREE. Black circles on nodes show bootstrap support of 100. See Figure 2 for more detailed phylogenetic information. Boxes surrounding pie charts show individuals sampled at the same locality.

Landscape evolution mediated by climate change appears to be partially responsible for the great diversity of freshwater mussels (Order Unionida), with over 220 species endemic to eastern North America (Graf and Cummings 2021; Simpson 1900). In addition to high species richness and endemism in North America, freshwater mussels are critically imperiled, with over 40% of the extant fauna listed or petitioned for listing under the US Endangered Species Act (ESA) (Hopper et al. 2023). For imperiled invertebrate taxa like freshwater mussels, the ESA does not recognize evolutionary significant units (ESUs), making accurate species (and subspecific) delimitation necessary for ESA protection (National Research Council 1995). The Mapleleaf freshwater mussel, *Quadrula quadrula* (Rafinesque 1820), formerly comprised three species: *Q. apiculata* (Say 1829), found in southern rivers draining into the northern Gulf of Mexico from Western Gulf drainages to the Mobile Basin (presumably introduced into the Rio Grande and Tennessee River of the Mississippi drainage; Howells, Neck, and Murray 1996; Parmalee and Bogan 1998), *Q. quadrula* sensu stricto (*s. s*.), found in the Great Lakes, Hudson Bay, and Mississippi drainages, and *Q. rumphiana* (Lea 1852), found exclusively in the Mobile Basin (Howells, Neck, and Murray 1996; Parmalee and Bogan 1998; Watters, Hoggarth, and Stansbery 2009; Williams, Bogan, and Garner 2008). In addition to geography, species were distinguished by notable morphological differences: *Q. apiculata* possessing small tubercles uniformly covering the external shell surface with a weak or absent sulcus (i.e., depression), *Q. quadrula s. s*. with two rows of tubercles on either side of the sulcus, and *Q. rumphiana* with a strong posterior ridge running alongside the sulcus and less organized but larger tubercles than *Q. apiculata* (Figure 1A). Previous work using molecular phylogenetics of female‐inherited mitochondrial (F‐mtDNA) markers found *Q. quadrula s. s*. paraphyletic with respect to *Q. apiculata* and *Q. rumphiana*, resulting in the collapse of the complex to a single taxon, *Q. quadrula* (Lopes‐Lima et al. 2019; Serb, Buhay, and Lydeard 2003). Morphological variation was explained as regional intraspecific variation despite overlapping distributions of phenotypic variants (Lopes‐Lima et al. 2019).

The *Q. quadrula* species complex has a fascinating life history. During the early life stage, specialized larvae, called glochidia, are temporary obligate parasites of fish. Brooding females in the tribe Quadrulini display an extension of their mantle, called a mantle magazine, that temporarily stores glochidia. The mantle magazine is understood to function as a lure to attract fish hosts by mimicking prey items (Barnhart, Haag, and Roston 2008; Sietman, Davis, and Hove 2012). Through predatory attacks on the mantle magazine, glochidia are extracted from the female, where they attach and become encysted on the gills of Channel Catfish (*Ictalurus punctatus*), Blue Catfish (*Ictalurus furcatus*), and possibly Flathead Catfish (*Pylodictis olivaris*) (Howard and Anson 1922; Schwebach et al. 2002; M. Hove, Univ. of Minnesota, written comm. 2024). Catfish hosts of *Q. quadrula* aid in their dispersal, growth, and metamorphosis into the free‐living juvenile life stage (Hove et al. 2012; Howard and Anson 1922). Perhaps owing to host‐mediated dispersal and the mobility of their catfish hosts, populations of *Q. quadrula* have been shown to colonize newly available habitats quickly, which may contribute to their widespread distribution (Hoffman, Morris, and Zanatta 2018; Parmalee and Bogan 1998; VanTassel et al. 2020).

Correlations between morphology and geography, and in particular overlapping zones among *Q. quadrula* phenotypic variants, present an ideal system to test the extent of reproductive isolation among potentially shallowly diverged lineages. Previously reported paraphyletic mtDNA clades may be the result of multiple mechanisms, but most expected are incomplete lineage sorting and introgression. In early diverging lineages, ancestral polymorphisms will persist in proportion to the effective population size, causing incongruences between mtDNA gene trees and species divergence (McKay and Zink 2010). A lack of correlation between geography and mtDNA haplotypes is evidence for incomplete lineage sorting (Toews and Brelsford 2012). Alternatively, introgressive hybridization creates populations with mixed ancestry within contact zones. Introgressed mtDNA haplotypes may persist outside of contact zones but at lower frequencies. Freshwater mussels possess a unique mitochondrial inheritance mechanism where females transmit a F‐type mitochondrial genome to all offspring and males transmit an independent M‐type mitochondrial genome stored in gonadal tissues exclusively to male offspring, called doubly uniparental inheritance (DUI) (Breton et al. 2007; Hoeh et al. 1996). Due to differences in the effective population sizes of F‐type and M‐type and the rate of evolution (Liu, Mitton, and Wu 1996; Zouros 2013), analysis of both mitochondrial genomes alongside nuclear DNA may be particularly effective in identifying incomplete lineage sorting or introgression for documented phylogenetic, phenotypic, and geographic discordances, which is fundamental to delineating species boundaries and conservation management of this imperiled group.

Here, we test the hypothesis that formerly recognized *Q. apiculata* and *Q. rumphiana* represent independently evolving lineages separate from *Q. quadrula*. We use extensive geographic sampling with a particular focus on areas of overlapping distributions, such as the Mobile Basin, combined with molecular sequencing from three separately evolving genomes, including female‐ and male‐inherited mitochondrial markers and thousands of nuclear single nucleotide polymorphisms (SNPs). We combine molecular markers with distributional data and conchological identifications for an integrative and comparative dataset, which we use to (1) identify the mechanisms of phylogenetic‐morphological discordances discovered in previous studies, (2) diagnose the demographic and divergence history of *Q. quadrula*, and (3) delineate the number of independently evolving lineages in the *Q. quadrula* complex.

## 2 Materials and Methods

### 2.1 Specimens

We examined 164 specimens from the *Q. quadrula* species complex through field‐collection or museum loan (Table S1). Field collected mussels were obtained through wading, snorkeling, and SCUBA. Our sampling emphasized known contact zones where previous studies have identified phylogenetic (Serb, Buhay, and Lydeard 2003; Lopes‐Lima et al. 2019) and morphological diversity (Howells, Neck, and Murray 1996; Neel 1941; Williams, Bogan, and Garner 2008). We also include samples collected near the type localities of *Q. quadrula, Q. rumphiana*, and *Q. apiculata* (Figure 1A). Six experienced field malacologists identified specimens to species groups based on shell characters listed in species descriptions (Rafinesque 1820; Say 1829; Lea 1852; Howells, Neck, and Murray 1996; Watters, Hoggarth, and Stansbery 2009; Williams, Bogan, and Garner 2008), including shape, tubercle pattern and size, external ray pattern, sulcus depth and width, and extension of the posterior ridge. Identifications were made from photographs that included coarse locality information (e.g., Mobile Basin). Identifications were independent from each other and molecular data. See Keogh and Johnson (2024) for all data, including specimen identifications and photographs, as well as scripts to repeat analyses described below.

### 2.2 Sanger Sequencing and Analyses

Our Sanger dataset consisted of two protein‐coding genes from female mitochondrial DNA (F‐mtDNA): *cytochrome c oxidase subunit I* (COI‐F) and *NADH dehydrogenase subunit I* (NDI), one nuclear‐encoded ribosomal locus: *internal transcribed spacer I* (ITSI), and one protein‐coding gene from male mitochondrial DNA (M‐mtDNA): *cytochrome c oxidase subunit I* (COI‐M), which are all commonly used for unionid systematics (e.g., Doucet‐Beaupré et al. 2012; Keogh and Simons 2019; Porto‐Hannes et al. 2021; Smith et al. 2018). For amplification of COI‐F, NDI, and ITSI, we extracted DNA from the foot or mantle tissue using the Qiagen DNAeasy Blood and Tissue Kit and polymerase chain reaction (PCR). Primers and temperature profiles were consistent with Folmer et al. (1994; COI‐F), Serb et al. (2003; NDI), and King et al. (1999; ITSI). For amplification of COI‐M, we extracted DNA from gonadal tissue within the visceral mass and designed our own primers (242F: 5′‐CAC CTC AGG ATG CCC AAA AA‐3′ and 894R: 5′‐GGG GTT TGG TTG GTT TGT CT‐3′) based on the published male mitogenome of *Q. quadrula* (NCBI: FJ809751.1; Breton et al. 2009) in Geneious v6.1.6 and ran PCR for 35 cycles (94C:1 min, 49C:1 min, 72C:1.5 min). All PCR reactions were 12.5 μL in volume: 1.5 μL DNA, 2.75 μL water, 6.25 uL Green Master Mix (Promega), and 1.0 μL of each primer. We added an additional 96 COI‐F sequences (NCBI: KX853887‐KX853982) from Mathias et al. (2018), which had greater sampling from the Great Lakes and Ohio River drainages. Morphology could not be assessed for these samples.

Bidirectional Sanger sequencing was performed at the University of Minnesota Genomics Center, the University of Florida, and the Smithsonian Institution. Chromatograms were trimmed, assembled, and edited in Geneious v6.1.6 and aligned using the MUSCLE algorithm (Edgar 2004) in AliView (Larsson 2014). Female‐ and male‐inherited mitochondrial genes were aligned with published *Q. quadrula* male and female mitochondrial genomes (NCBI: FJ809750.1 & FJ809751.1; Breton et al. 2009) to ensure successful amplification and codon position. Additionally, we sampled outgroup species *Q. fragosa* (*n* = 18, swabs only: U.S. Fish & Wildlife permits: TE206778‐7 & TE206781‐9), *Q. nobilis* (*n* = 7), *Q. verrucosa* (*n* = 3), and *Cyclonaias tuberculata* (*n* = 1).

We ran PartitionFinder v1.1.1 (Lanfear et al. 2012) on our F‐mtDNA (COI‐F & NDI) and M‐mtDNA (COI‐M) datasets to determine substitution models for each codon position (COI‐F: K80+G, HKY, HKY+I; NDI: HKY+I, HKY+G, K80+I; COI‐M: HKY, HKY+I, HKY+I). Using these datasets, we ran two BEAST v2.6.6 (Bouckaert et al. 2019) analyses (one for each mtDNA dataset) in the CIPRES Science Gateway (Miller et al. 2015). We used a strict clock model and a birth death tree prior, ran a MCMC of 100,000,000 generations sampling every 5000, visualized log files in Tracer v1.7.1 (Rambaut et al. 2018), combined three runs in LogCombiner v2.6.6, generated a maximum clade creditability tree, and assessed a 30%–40% burnin in TreeAnnotator v2.6.6 (Bouckaert et al. 2019) for each analysis. In addition to the BEAST analyses, we ran RAxML v8.2.12 on the concatenated F‐mtDNA dataset for a maximum likelihood phylogenetic inference in CIPRES (Stamatakis 2014). We created TCS haplotype networks of COI‐M and ITSI in PopArt (Leigh and Bryant 2015).

### 2.3 RAD Sequencing and Assembly

A subset of the specimens described above were selected for a modified method of genomic dual‐digest restriction‐site‐associated DNA (ddRAD) sequencing called 3RAD (Bayona‐Vásquez et al. 2019). We selected specimens that maximized mitochondrial, morphological, and geographic diversity as well as individuals from all outgroup species. We used DNA extractions taken from the foot or mantle tissue. We quantified and normalized DNA samples to 20 ng/μL using a Qubit Fluorometer and SpeedVac. Two libraries were prepared following the Adapterama III protocol (Bayona‐Vásquez et al. 2019): one at the RadCamp 2019 workshop (radcamp.github.io/NYC2019) and a second at the EHS DNA Laboratory at the University of Georgia (UGA). Samples were digested with enzymes XbaI, EcoRI‐HF, and NheI, followed by iTru adapter ligation of variable length internal indexes. Following digestion and ligation, samples were pooled and purified using SpeedBeads. PCR was then used to amplify 3RAD libraries with iTru5 and iTru7 primers (Glenn et al. 2019). Products were cleaned again using SpeedBeads and quantified using a Qubit Fluorometer. A Pippin Prep (Sage Science) was used to size select for 525 bp ± 10% fragments. The RadCamp library was pooled with unrelated projects and sent to Admera Health for Illumina sequencing on a HiSeq X platform for a read depth of 40 million with PE150 reads. The UGA library was sequenced at Novagene Corporation on a HiSeq X with PE150 reads.

Raw 3RAD reads were first inspected for quality in FastQC v0.11.9 (Andrews 2010). We assembled reads in ipyrad v0.9.68 (Eaton and Overcast 2020) with default settings aside from the following modifications: assembly method set to “denovo,” datatype “pair3rad,” restriction overhang was “GCTAG, TAATTC” for the RadCamp library and “CTAGA, AATTC” for the UGA library, maximum barcode mismatch was “1,” clustering threshold was set to “0.93”, and the minimum number of samples per locus (missing data parameter) was set to “7” (90% missing), “17” (78% missing), “34” (56% missing), and “51” (34% missing) to create four datasets, and filtered out all reads that mapped to the male and female mitochondrial genomes of *Q. quadrula* (NCBI: FJ809750.1 & FJ809751.1; Breton et al. 2009) to ensure the RAD dataset contained only nuclear loci (no reads were ultimately mapped to reference genomes). We merged the RadCamp and UGA data after step 1 of the ipyrad pipeline. We explored other datasets created through different clustering thresholds, including 0.85, 0.90, 0.95, and 0.97; however, we chose 0.93 because the dataset had the best tradeoff of total SNPs, heterozygosity, error rate, and cumulative variation in the first eight principal components (Figure S1; McCartney‐Melstad, Gidiş, and Shaffer 2019). See Table S2 for ipyrad output files used in analyses.

### 2.4 Phylogenomics and Population Structure

We used our 3RAD datasets to construct phylogenies with IQ‐TREE 2 (Minh et al. 2020). We used concatenated loci from all four 3RAD datasets, including 67 individuals from the *Q. quadrula* complex and 10 individuals total from all outgroup species. We implemented ModelFinder (Kalyaanamoorthy et al. 2017) within IQ‐TREE to find substitution models based on the Bayesian information criterion (Table S2). Topological support was acquired using 1000 ultrafast bootstraps (Hoang et al. 2018), and consensus trees were visualized and rooted at the midpoint in FigTree v1.4.4 (Rambaut et al. 2018). Because subsequent population structure analyses showed evidence of admixture in samples from the Mobile Basin, we made an additional ipyrad assembly without these admixed samples (“nomob”) and ran IQ‐TREE to assess the influence these samples had on tree topology.

We found the *Q. quadrula* complex to be monophyletic in all phylogenetic analyses and therefore created new 3RAD datasets in ipyrad with outgroup taxa excluded (“ingroup” assembly, *n* = 67). We calculated ancestor coefficients and assigned individuals to groups using two cluster‐based analyses. For both analyses, we used one random SNP per locus from the “ingroup” assembly (~24% missing data). First, we used a sparse non‐negative matrix factorization (sNMF) method implemented in the R package *LEA* (Frichot and François 2015). We estimated ancestry coefficients for 1–8 ancestral populations (*K*) with 20 replicates per *K* for 100,000 iterations. The number of ancestral populations, *K*, was selected based on the cross‐entropy criterion (Frichot et al. 2014). We used the *impute* function to input missing data based on the most likely genotype (method = ‘mode’). Second, we used a Bayesian approach to visualize population structure with STRUCTURE v2.3.4 (Pickrell and Pritchard 2012) through the ipyrad API (Eaton and Overcast 2016). We filtered SNPs that occurred in less than 90% of samples and conducted three replicates for each *K* (2–5). All MCMC chains were run for 125,000 generations with a 25,000 burnin. We selected *K* based on the deltaK criterion (Evanno, Regnaut, and Goudet 2005). Both sNMF and STRUCTURE analyses indicated four ancestral populations: cross‐entropy scores reached a minimum at *K* = 4, and deltaK peaked at *K* = 4. In addition to *K* = 4, we explored ancestry coefficients using three and five ancestral populations using the *sNMF* function in *LEA*. Missing data was imputed separately for each *K*. Many downstream analyses require *a priori* identification of samples to putative species. We therefore used the ancestry coefficients from the sNMF analysis to assign samples to four groups, consistent with cross‐entropy criterion. Because sNMF ancestry coefficients were congruent with coefficients recovered from STRUCTURE and broadly concordant (but with overlap) with morphological identifications and F‐mtDNA, groups were named after recognized or previously recognized *Quadrula* species: “apiculata‐East,” “apiculata‐West,” “quadrula,” and “rumphiana”.

We visualized genomic diversity through principal component analysis (PCA) with the ipyrad API (Eaton and Overcast 2016). We used *K*‐means clustering to impute missing genotypes, assuming *K* = 4, as determined by STRUCTURE and sNMF analyses. *K*‐means clustering iteratively assigns samples to groups and then imputes missing data based upon the allele frequencies of the assigned group. Therefore, imputation is robust to *a priori* population assignments (https://ipyrad.readthedocs.io/en/latest/API‐analysis/cookbook‐pca.html). We used five iterations, starting with SNPs shared across 90% of samples and progressively allowing more missing data until we reached SNPs present for only 60% of samples. We then ran the PCA subsampling one random SNP per locus for 100 replicates. This procedure was conducted twice. One to visualize genomic diversity of *Q. quadrula* groups (“apiculata‐East”, “apiculata‐West”, “quadrula”, and “rumphiana”) and another to visualize diversity between the two 3RAD library preps/sequencing runs (RadCamp and UGA).

### 2.5 Gene Flow

Consistent with the literature (Lopes‐Lima et al. 2019; Williams, Bogan, and Garner 2008), we found the Mobile Basin to be a contact zone that contains samples with “apiculata‐East” and “rumphiana” ancestry and morphologically identified as *Q. apiculata* and *Q. rumphiana*. Given the importance of contact zones in species delimitation (Chambers, Marshall, and Hillis 2023), particularly in understanding the extent of introgression between lineages, we examined gene flow in the Mobile Basin using two approaches. We used the R package *gghybrid* to infer the strength of gene flow between “apiculata‐East” and “rumphiana” populations through the hybrid index using 3000 MCMC generations, with 1000 generations discarded as burnin (Bailey 2022). We used one random SNP per locus and removed all samples outside of the Mobile Basin aside from three samples from the Pascagoula River, the drainage immediately east of the Mobile Basin. Because we failed to find any “apiculata‐East” with pure ancestry within the Mobile Basin, we used the Pascagoula individuals as parental samples and three “rumphiana” individuals with at least > 95% ancestry as the other parental group to characterize heterozygosity within the Mobile Basin for SNPs fixed between parental groups.

We used the same parental groupings to estimate the extent of hybridization. If admixed individuals in the Mobile Basin are primarily composed of second‐generation (F2s) and backcrossed individuals, this would be evidence against reproductive isolation. We used newhybrids (Anderson and Thompson 2002), which uses MCMC to calculate probabilities of six genotype classes: pure “apiculata‐East,” pure “rumphiana,” first‐generation hybrid (F1), second‐generation hybrid (F2), backcrossed “apiculata‐East,” and backcrossed “rumphiana.” We extracted one biallelic SNP per locus in VCFtools v0.1.16 (Danecek et al. 2011). We used the R package *dartR* (Gruber et al. 2018) to extract SNPs with a call rate > 90%, remove monomorphic SNPs, find 200 loci fixed between parental populations, and run newhybrids (*gl.nhybrids* function) for 10,000 sweeps.

We tested for isolation by distance using genetic distance (F_ST_) and Euclidean distance of geospatial coordinates through a Mantel test in the R package *dartR* (Gruber et al. 2018). We manipulated the VCF file from ipyrad to remove sites that were non‐biallelic, linked, and had minor allele counts < 1 in VCFtools v0.1.16 (Danecek et al. 2011). We ran a total of three analyses. First, we used only Pearl, Pascagoula, and Mobile Basin samples to assess isolation by distance for the “rumphiana” and “apiculata‐east” groups. Second, we used all 67 ingroup samples to test for isolation by distance across *Q. quadrula*. For the first two analyses, sampled localities consisting of one sample were pooled with populations within 250 km of contiguous river distance to increase population sizes, but with two exceptions: a Colorado River sample was pooled with the neighboring Navidad River population and a Pearl River sample was pooled with the Pascagoula River population. These first two analyses do not account for the strictly aquatic dispersal of *Q. quadrula*. Population genetic studies on aquatic taxa have used river distance when testing for isolation by distance, but river distance fails to account for historical stream capture events and is thus best used for assessing within‐drainage distances (Crookes and Shaw 2016; Johnson et al. 2023; Seidel, Lang, and Berg 2009). To try to account for geographic barriers to gene flow such as drainage divides and seawater, we used the centroid of each isolated river drainage (a contiguous river network that drains into the ocean) and clustered all samples collected from each drainage to their centroid. This method assumes populations within drainages are more similar to each other than to populations from other drainages. To calculate centroids, we downloaded the Watershed Boundary Dataset from the United States Geological Survey and used the R packages *rgeos* and *rgdal* (USGS 2020; Bivand and Rundel 2023; Bivand, Keitt, and Rowlingson 2023).

### 2.6 Species Tree

We used the multispecies coalescent framework to estimate a time‐calibrated species tree of the *Q. quadrula* complex with the SNAPP package (Bryant et al. 2012) in BEAST 2.6.6 (Bouckaert et al. 2019). To reduce computational time, we included 28 ingroup samples and one outgroup species, *Q. fragosa* (3 samples). We used a strict molecular clock and set a uniform prior to the crown age of *Q. fragosa* and the *Q. quadrula* complex set between 9 and 12 Ma. This calibration prior is consistent with Neemuchwala et al. (2023), who used fossil and biogeographic evidence to time‐calibrate the Quadrulini phylogeny. We used the results of the sNMF analysis to assign ingroup samples to populations (*K* = 4: “apiculata‐East,” “apiculata‐West,” “quadrula,” and “rumphiana”). Because we found evidence of gene flow between ingroup samples and this species tree method assumes no gene flow, we removed samples that showed evidence of mixed ancestry (< 0.95 ancestry coefficient) aside from one “apiculata‐East” sample to ensure each putative species consisted of samples from more than one locality. We used the R package *phrynomics* (http://github.com/bbanbury/phrynomics) to remove nonbinary and invariant sites. We then used the ruby script from https://github.com/mmatschiner/snapp_prep to generate the SNAPP input file (Stange et al. 2018) and conducted three independent analyses for 500,000 generations each, sampling every 250 generations. Log files were examined in Tracer (Rambaut et al. 2018) to ensure proper mixing, convergence of runs, and high effective sample sizes. Tree files were combined in LogCombiner v2.6.6, and a maximum clade creditability tree was generated with 20% burnin assessed in TreeAnnotator v2.6.6 (Bouckaert et al. 2019).

### 2.7 Species Delimitation and Demography

Given evidence of gene flow between putative species, species delimitation methods that assume a strictly divergence‐only speciation process were not used (e.g., multispecies coalescent framework; Rannala and Yang 2003). Instead, we used the R package *delimitR* (Smith and Carstens 2020), which incorporates demographic processes including divergence with ongoing gene flow and secondary contact when estimating species boundaries. *DelimitR* uses fastsimcoal v2.6 (Excoffier et al. 2021) to simulate user‐defined evolutionary models in the form of multidimensional site frequency spectra. Then random forest machine learning is trained on the simulated data and applied to the observed data to select the user‐defined speciation model(s) whose simulated data best matches the observed data (Peede, D'Agostino, and Ottenburghs 2020; Smith et al. 2017). We generated a site frequency spectrum (SFS) from the ingroup assembly using easySFS (https://github.com/isaacovercast/easySFS). We removed non‐biallelic and linked sites in VCFtools v0.1.16 (Danecek et al. 2011), monomorphic sites in BCFtools v1.10.2 (Danecek et al. 2021), and invariant sites in R packages *SNPfiltR* (DeRaad 2022) and *vcfR* (Knaus and Grünwald 2017). We down sampled SNPs and individuals to account for missing data to 18 diploid “apiculata‐East” samples, 7 diploid “apiculata‐West” samples, 13 diploid “quadrula” samples, and 11 diploid “rumphiana” samples for a total of 10,201 SNPs. We constructed 26 speciation scenarios in fastsimcoal v2.6, including single species, two species, three species, and four species models, with a tree topology consistent with the SNAPP analysis. Up to four migration events were specified, and migration was allowed between all populations aside from between “rumphiana”‐“apiculata‐West” and “rumphiana”‐“quadrula,” as we found no evidence of gene flow between these groups. Broad priors for divergence time (40,000–150,000 generations before present), population size (10,000–1000,000 haploid individuals), and migration rate (0.05–50 migrants/generation) were informed by previous studies including divergence time estimates of *Quadrula* species (Neemuchwala et al. 2023) and other closely related freshwater mussels occupying similar ranges (Inoue et al. 2020; Pieri et al. 2018) as well as the estimated generation time of *Q. quadrula* (Whitney, Blodgett, and Sparks 1997) and closely related species (Haag and Staton 2003; Fobian 2007). We simulated 10,000 multidimensional site frequency spectra for all 26 models and summarized simulated and observed data into six bins per population. We used 1000 decision trees to build the random forest classifier. See Figure S11 for the full set of tested models.

**FIGURE 2:**
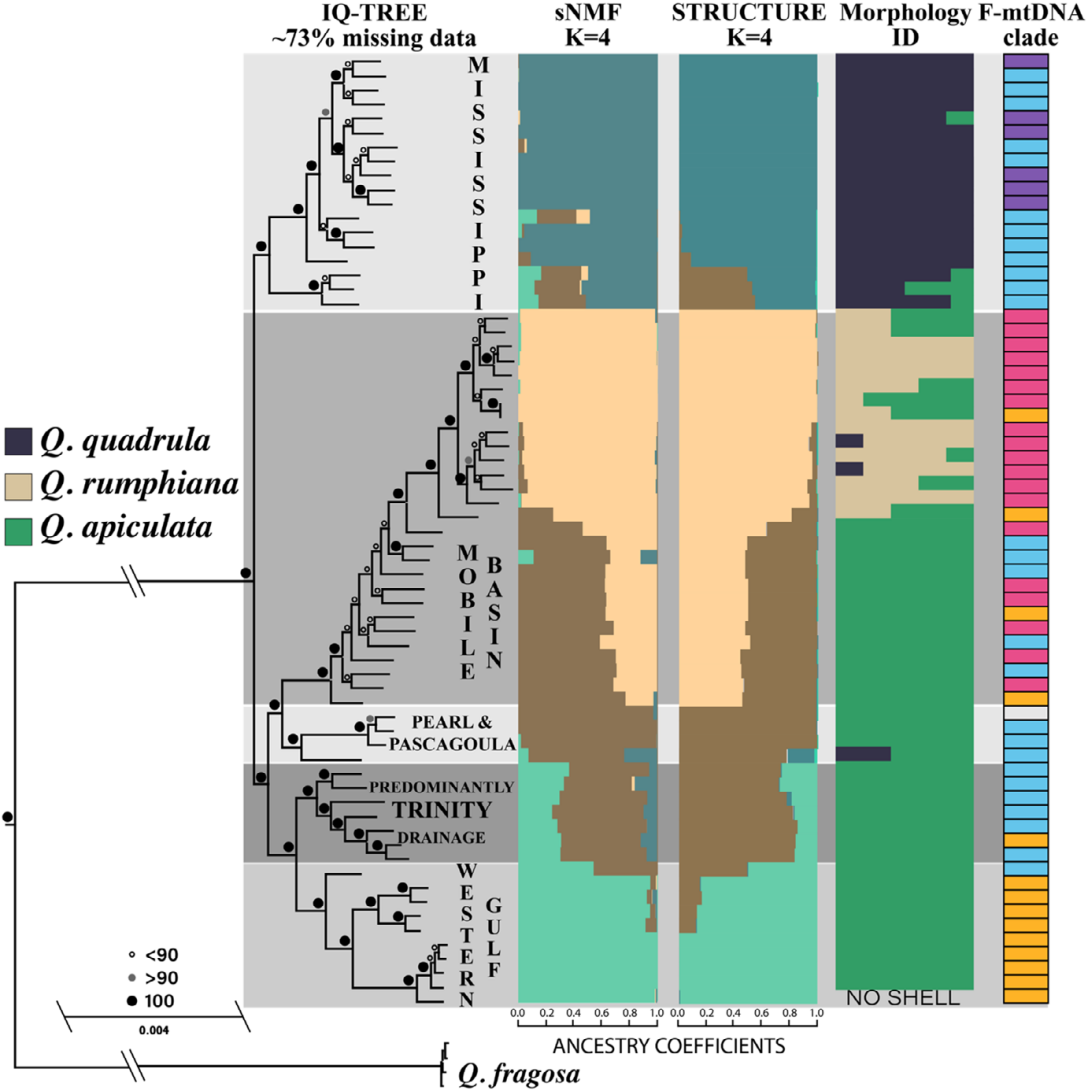
IQ‐TREE phylogeny constructed from the ‘msl17’ SNP dataset alongside ancestry coefficients produced by sNMF and STRUCTURE analyses. The ‘Morphology ID’ bar plot shows how each specimen was identified by six malacologists, and the F‐mtDNA column shows F‐mtDNA clade membership. Color schemes are consistent with Figure 1. White color for F‐mtDNA clade membership shows no data for that specimen.

Because results from the above ‘four‐species’ analysis strongly favored all four groups as distinct species, we created an expanded ‘six‐species’ analysis to test if *delimitR* could distinguish between intraspecific geographic variation and independently evolving species. We expanded the “apiculata‐East” group into three groups: Mobile Basin, Pearl & Pascagoula, and Trinity, representing geographic structure recovered in sNMF and IQ‐TREE analyses. After samples were assigned to groups, we used the same vcf file to down sample SNPs and individuals using easySFS (4 diploid Mobile Basin samples, 3 Pearl & Pascagoula, 4 Trinity, 5 “quadrula,” 5 “apiculata‐West,” and 6 “rumphiana”; https://github.com/isaacovercast/easySFS). In total, we constructed 52 models of speciation, allowing up to two migration events per model. We used the SNAPP phylogeny with the “apiculata‐east” group broken into three subpopulations, with Mobile Basin and Pearl & Pascagoula being more closely related to each other than the Trinity River group, consistent with IQ‐TREE analyses (Figures 2 and S6). Otherwise, the same parameters were chosen with exception to a wider prior divergence time to accommodate the additional bifurcations (5000–150,000 generations before present), four bins were used to summarize the site frequency spectra, and 500 decision trees were used to build the random forest classifier.

## 3 Results

### 3.1 Morphology and Sanger Analyses

Consensus morphological identifications of specimens revealed partitioning of phenotypes into mostly distinct geographic zones (Figure 1A). Specimens from the Mississippi drainage were identified as *Q. quadrula s. s*. with exception to six specimens (no species identification was in the majority). Specimens from the Western Gulf, Trinity & Sabine, and Pearl & Pascagoula geographic zones were all identified as *Q. apiculata* aside from one specimen from the Trinity & Sabine identified as *Q. quadrula s. s. Quadrula rumphiana* was identified from localities exclusively within the Mobile Basin, along with high numbers of *Q. apiculata*. The Mobile Basin was the only geographic zone containing specimens identified as different species at multiple localities.

The Bayesian and maximum likelihood molecular phylogenies generated from female‐inherited mitochondrial markers, COI‐F and NDI, recovered four major clades with congruent topologies of the *Q. quadrula* complex (Figures 1B and S2). There was a clear latitudinal gradient consisting of individuals from the most divergent clade at northern latitudes (Mississippi and Great Lakes regions) to individuals from the second most divergent clade in the south of the Mississippi drainage. Samples from this F‐mtDNA clade occurred in all other geographic zones (aside from the Great Lakes) but were most prevalent in the southern Mississippi drainage and areas immediately east and west. Individuals from the Western Gulf were predominately recovered in a single clade, with clade members extending east into the Trinity & Sabine and Mobile Basin. Samples from the fourth clade were found exclusively within the Mobile Basin. For the male‐inherited mitochondrial marker COI‐M, the Bayesian phylogeny and TCS haplotype network showed some similar phylogeographic patterns to F‐mtDNA phylogenies, with one haplotype cluster found exclusively in the Mobile Basin, another found exclusively in the Mississippi drainage, and other clusters sampled from multiple drainages (Figures 1C and S3). However, F‐mtDNA phylogeny, morphology, and M‐mtDNA haplotype data were not congruent (Figure S4). For example, *Q. quadrula s. s*. identified individuals had similar M‐mtDNA haplotypes, but they occurred in two separate F‐mtDNA clades. The nuclear marker ITSI was mostly invariable (Figure S5).

### 3.2 Phylogenomics and Population Structure

All IQ‐TREE analyses using the 3RAD SNPs dataset recovered the *Q. quadrula* complex as monophyletic and sister to *Q. fragosa* (Figures 2 and S6). Only one analysis found *Quadrula* to be monophyletic (Figure S6B), but greater outgroup sampling is needed to test the monophyly of *Quadrula*. Within the ingroup, samples from the Mobile Basin formed a clade sister to samples from the Pearl & Pascagoula drainages (southeastern clade). This southeastern clade was sister to a southwestern clade containing samples from the Trinity drainage and Western Gulf. The greater southeastern + southwestern clade, containing samples from isolated Gulf of Mexico coastal drainages, was sister to a clade from the greater Mississippi Basin. The one IQ‐TREE analysis that differed was based on the dataset containing no admixed Mobile Basin samples. In this case, the Mississippi drainage clade was sister to a clade containing Pearl & Pascagoula, Western Gulf, and Trinity drainage samples. The Mobile Basin clade was recovered as the earliest diverging and sister to all other ingroup samples (Figure S6D).

**FIGURE 3:**
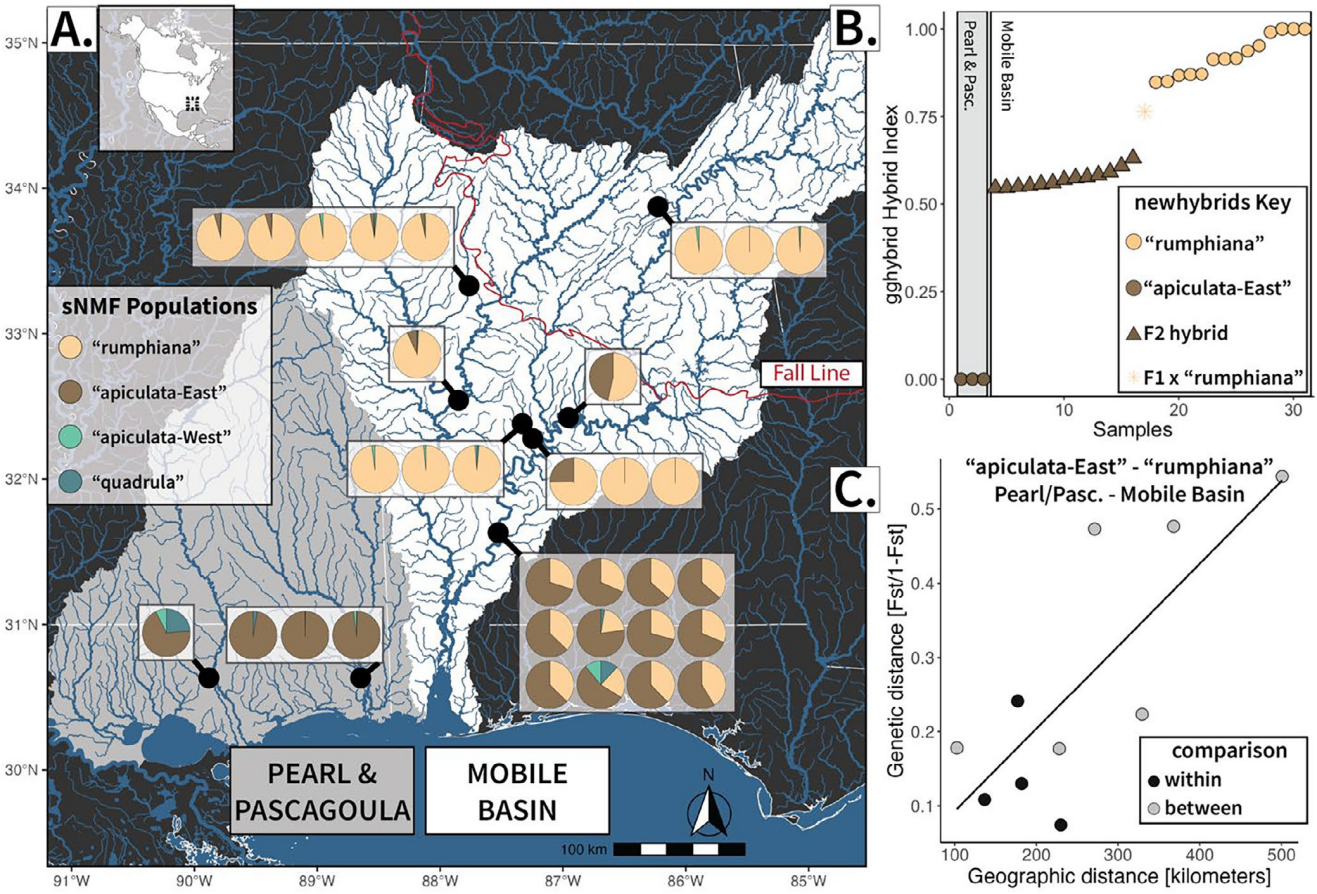
(A) Collection localities of samples from contact zone Mobile Basin and neighboring Pearl & Pascagoula drainages. Each pie chart shows the sNMF ancestry proportions of a single individual per sampled locality (boxes). (B) Hybrid indices calculated from *gghybrid* and hybrid classifications calculated from newhybrids using SNPs for all ingroup Mobile Basin samples. Three samples collected from the Pascagoula River with high “apiculata‐East” ancestry and three samples with the highest “rumphiana” sNMF ancestry were used as parental samples. Points are colored by the majority sNMF ancestry. Point shapes show calculated hybrid classification from newhybrids. No first‐generation hybrids (F1) or F1 × “apiculata‐East” were identified, and therefore they do not appear in the key. (C) Isolation by distance plot of SNPs data showing pairwise comparisons between sampled populations in the Pearl‐Pascagoula drainages and Mobile Basin (pictured in A). Comparisons between putative lineages “apiculata‐East” and “rumphiana” are colored gray, and comparisons within lineages are colored black. Code from Poelstra et al. (2021) was helpful in visualizing results.

Ancestry coefficients between sNMF and STRUCTURE analyses were similar per sampled individual at *K* = 4 (Figure 2). Samples collected from the Mississippi River drainage were overwhelmingly identified morphologically as *Q. quadrula s. s*. and had majority ancestry from a single group, which we refer to as “quadrula” hereafter (Figure 1D). The Mobile Basin contained samples with ancestry from two groups (Figures 2 and 3A). Samples with pure ancestry were mostly identified as *Q. rumphiana* (“rumphiana” group hereafter). However, samples identified as *Q. apiculata* had mixed ancestry between pure samples from the Mobile Basin and pure samples from the Pearl & Pascagoula drainages (“apiculata‐East” group hereafter). A gradient in ancestry coefficients also spanned from pure Pearl & Pascagoula samples to mixed ancestry in the Trinity drainage to pure ancestry in the Western Gulf (Figure 1D). This gradient in ancestry also follows a geographic gradient from east to west with the Trinity River between the Pearl & Pascagoula drainages (east) and the Western Gulf (west). As all samples from these drainages were identified as *Q. apiculata*, samples from the Western Gulf are referred to as “apiculata‐West” hereafter. When three ancestral populations were considered, pure ancestry in the Western Gulf spanned the Trinity and Pearl & Pascagoula drainages, where it was mixed with Mobile Basin ancestry for half the Mobile Basin samples. The other half of Mobile Basin samples had unique ancestry. The Mississippi drainage contained a unique population that was mixed with the Western Gulf ancestry for a few samples. At *K* = 5, the results were similar to *K* = 4, but with the mixed‐ancestry Mobile Basin samples split into a unique population (Figure S7).

The principal component analysis (PCA) of the 3RAD SNPs dataset closely matched the ancestry analyses (Figure S8). Along principal component (PC) 1 (22.6% of variation), there was a gradient between “rumphiana” samples and all other ingroup samples. Mobile Basin “apiculata‐East” samples clustered closer to “rumphiana” samples on PC 1. Along PC 2 (11.2% of variation), a gradient formed between “quadrula” and “apiculata‐West” samples with “rumphiana” and “apiculata‐East” samples between them. Variation along PC 3 produced a gradient from interior and northern samples (“apiculata‐East” and “quadrula”) to peripheral samples east and west (“rumphiana” and “apiculata‐West”). Along the first six principal components (46% of variation), we observed no genomic variation associated with separate sequencing runs (RadCamp and UGA; Figure S9).

### 3.3 Gene Flow

Hybridization results from *gghybrid* and newhybrids analyses identified “apiculata‐east” samples from the Mobile Basin as advanced hybrids (F2 or greater) with hybrid indices in between “apiculata‐east” parental samples from the Pascagoula River and “rumphiana” parental samples (Figure 3B). One “rumphiana” sample was identified as a F1 x “rumphiana” cross. Mapping of ancestry coefficients from the sNMF analysis within the Mobile Basin showed a north–south gradient between pure “rumphiana” samples in the northern Mobile Basin and admixed individuals in the south (Figure 3A). Isolation by distance analyses revealed positive relationships between genetic distance (F_ST_) and geographic distance for both the Pearl/Pascagoula‐Mobile Basin group (adjusted *R* ^2^ = 0.59, *p*‐value = < 0.001) and all ingroup samples (Euclidean: adjusted *R* ^2^ = 0.18, *p*‐value = < 0.001; clustered by drainage: adjusted *R* ^2^ = 0.05, *p*‐value = 0.02) (Figures 3C and S10).

### 3.4 Species Tree

**FIGURE 4:**
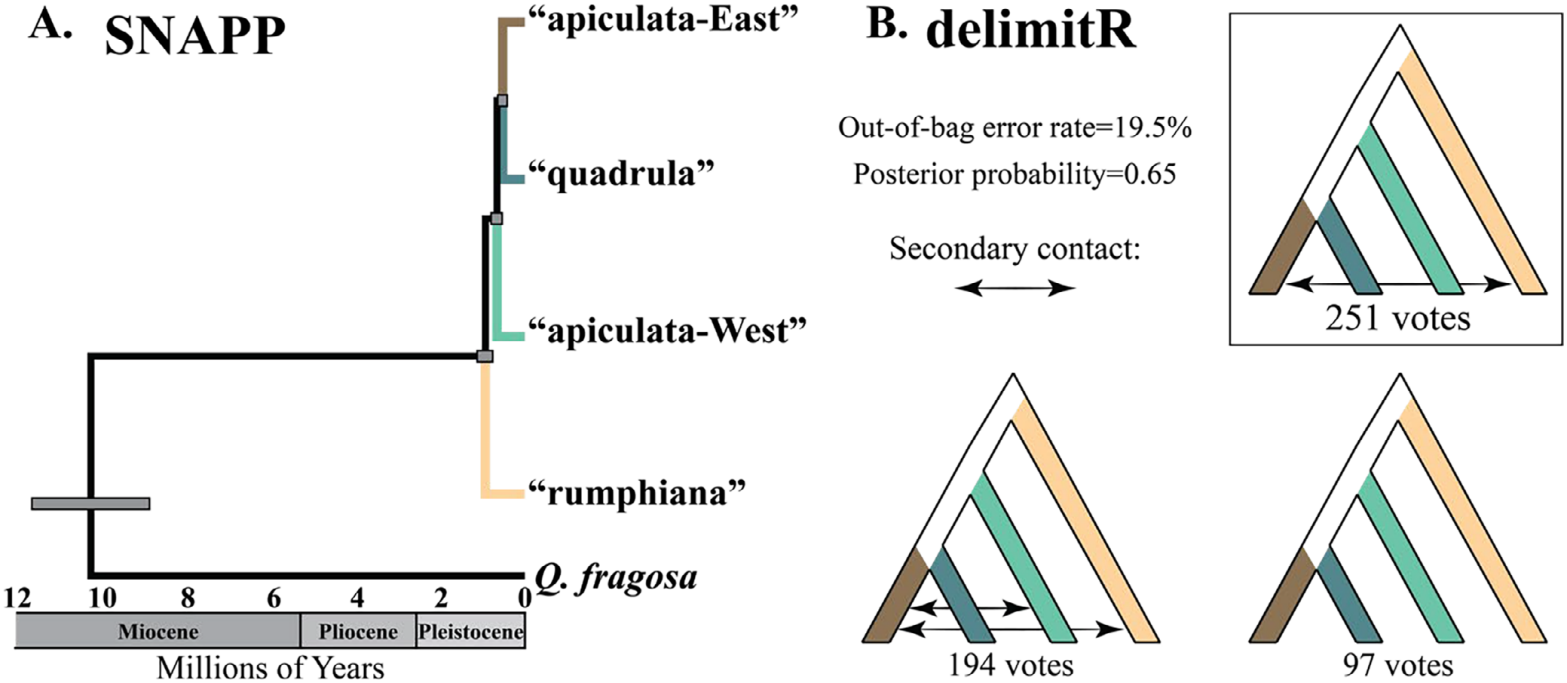
(A) Time‐calibrated SNAPP phylogeny of the *Q. quadrula* populations constructed from the SNPs dataset. Gray bars at nodes show 95% probability density for the interval of divergence times. All nodes had a posterior probability of 1.0. (B) The top three models recovered from the ‘four‐species’ *delimitR* analysis. Arrows indicate a model supporting secondary contact between tip populations. The number of votes shows model support. For a full list of models tested and their support, see Figure S11.

The SNAPP time‐calibrated species tree analysis recovered a fully supported tree with posterior probability values of 1.0 (Figure 4A). The root age of the *Q. quadrula* complex and *Q. fragosa* was estimated at 10.4 Ma (95% HPD = 9.0–11.8). The “rumphiana” lineage was the earliest divergence within the complex with a node age estimation of 0.95 Ma (95% HPD = 0.77–1.14), followed by the divergence of “apiculata‐West” at 0.67 Ma (95% HPD = 0.54–0.81). The “quadrula” and “apiculata‐East” lineages were sister to each other with an estimated divergence time of 0.53 Ma (95% HPD = 0.42–0.65).

### 3.5 Species Delimitation and Demography

For the ‘four‐species’ analysis, the best supported species delimitation model had all four groups recovered from STRUCTURE and sNMF analyses as distinct species (Figure 4B). Overall, 99.8% of votes supported a four species model (Figure S11). The top two performing models included secondary contact between “apiculata‐East” and “rumphiana,” two groups that are currently sympatric in the Mobile Basin, with “apiculata‐East” extending west to the Pearl‐Pascagoula region. In addition to this demographic event, the second‐best supported model included secondary contact between “apiculata‐East” and “apiculata‐West.” The overall out‐of‐bag error rate was 19.5% and posterior probability was 0.65. Results of the ‘six‐species’ analysis also strongly supported the maximum number of groups (six) as distinct species (Figure S12). The best supported model included secondary contact between the Trinity “apiculata‐East” and “apiculata‐West” and between the Mobile Basin “apiculata‐East” and “rumphiana” groups, results that were markedly similar to the second‐best supported model of the ‘four‐species’ analysis. The top five best supported models all included secondary contact between the Mobile Basin “apiculata‐East” and “rumphiana” groups. A cumulative 92.8% of votes supported six‐species models. The out‐of‐bag error rate was 38.46% and the posterior probability was 0.73.

## 4 Discussion

### 4.1 Species Delimitation and Conservation

We found *Q. quadrula*, a North American aquatic mollusk that ranges from the Great Lakes (as well as Lake Winnipeg) south to the Mississippi embayment and the Rio Grande east to the Mobile Basin, to exhibit strong spatial molecular structure associated with isolated river drainages that was continuous when variants met in contact and along adjoining drainage divides (Figure 1). Demographic analysis and phylogenetic divergence dating suggest these patterns are best explained by Pleistocene vicariance followed by secondary contact and gene flow, which has degraded historical phylogenetic and morphological diversification. These recurrent isolation‐contact events (i.e., fission‐fusion cycles; Musher et al. 2022; Maier et al. 2019; Meier et al. 2023), likely driven by Pleistocene climatic fluctuations and subsequent river rearrangement, may explain the incongruences across molecular (F‐mtDNA, M‐mtDNA, and nuDNA) and phenotypic characters (Figures 1 and S4). Although the best supported speciation scenarios contained each of the four tested lineages as distinct species, when considering all analyses holistically, there is little evidence that any of these four groups are independently evolving lineages (de Queiroz 2007). No group is monophyletic for both F‐mtDNA and nuDNA (SNPs) phylogenies (Figure 2); no group is free from genetic intermediates (Figures 1 and 2); the species complex as a whole follows a pattern of isolation by distance (Figures 3C and S10); and when groups are in contact, there is no evidence of reproductive isolation (Figure 3A,B). As such, we recommend no changes to the species‐level taxonomy of *Q. quadrula*.

These shallowly differentiated populations presented an ideal empirical system to test the accuracy of species delimitation tools. Despite strong clinal variation across the range of *Q. quadrula* and no evidence of reproductive isolation when in contact (Figure 3), *delimitR* strongly favored the maximum allowable populations as distinct species in both the ‘four‐species’ and ‘six‐species’ analyses (Figures 4B, S11, and S12). When considering species as independently evolving lineages, multispecies coalescent methods are prone to oversplitting species with highly structured populations (Chambers and Hillis 2020; Mason et al. 2020; Sukumaran and Knowles 2017). It appears that process‐based species delimitation methods, like *delimitR*, are also susceptible to delimiting intraspecific geographic variation as distinct species (Bamberger, Xu, and Hausdorf 2022; DeRaad et al. 2023). Of course, users need to be cognizant of the underlying assumptions of the method chosen when interpreting their results. As the developers of *delimitR* point out, given that gene flow is allowed between putative species, the decision to accept or reject delimited species will rest on how much gene flow is acceptable under the applied species concept (Smith and Carstens 2020). Nevertheless, for widespread, shallowly diverged lineages like those within *Q. quadrula*, geographic variation can lead to taxonomic overinflation by neglecting spatial gradients of phenotypic and genetic variation (Chambers, Marshall, and Hillis 2023; Marshall et al. 2021). As others have previously cautioned, the following are considerations when using species delimitation tools: (1) carefully design geographic sampling to emphasize geographically intermediate populations, ideally areas of contact; and (2) interpret species delimitation results holistically alongside phenotypic datasets and explicit tests of isolation by distance and gene flow (Cicero et al. 2021; Hausdorf and Hennig 2020; Hillis, Chambers, and Devitt 2021).

Even with scant evidence of independently evolving lineages, morphological forms of *Q. apiculata*, *Q. quadrula s. s*., and *Q. rumphiana* are generally congruent with nuDNA phylogenies and ancestry coefficients, with exception to the *Q. apiculata* variant split into an east and west population (Figure 2). These shell forms have been shown to be diagnosable but with clinal variation between drainages, which further supports the hypothesis of repeated episodes of isolation and contact (Lopes‐Lima et al. 2019; Neel 1941). The recognition of subspecies has historically been controversial (Burbrink et al. 2022; de Queiroz 2020; Hillis 2020) because designations infrequently reflect evolutionary lineages (e.g., MacGuigan et al. 2022). However, modern technology and analytical tools are now better able to identify inter‐lineage diversity, causing an uptick in delineating within‐species taxonomic diversity (Marshall et al. 2021; Natusch et al. 2020). Further, for critically imperiled groups like freshwater mussels, formal recognition of intraspecific diversity is necessary for their conservation. In contrast to vertebrates, ‘distinct population segments’ or evolutionary significant units (ESUs) are not recognized by the Endangered Species Act for invertebrates, and thus subspecific designations are paramount for conservation (National Research Council 1995). Our findings provide confirmation that “rumphiana” is endemic to the Mobile Basin and maintains genetic differentiation that corresponds to weakly identifiable morphological features. Our sampling and additional distributional datasets within the Mobile Basin show habitat segregation, as the “rumphiana” form is dominant in upstream habitats while “apiculata” is generally found in downstream lowland habitats (Pfeiffer, Dubose, and Keogh 2024; Williams, Bogan, and Garner 2008). We therefore recognize the “rumphiana” lineage as *Q. quadrula rumphiana* found in the Mobile Basin. Prior to its synonymy, *Q. rumphiana* was considered stable throughout the Mobile Basin (US states: Alabama, Georgia, Mississippi, and Tennessee; Garner et al. 2004). However, freshwater mussel population dynamics and viability can change quickly (e.g., Richard et al. 2020). Therefore, formal recognition of *Q. quadrula rumphiana* as a subspecies may facilitate future conservation and recovery planning if its conservation status changes. In addition to *Q. quadrula rumphiana*, names exist for other *Q. quadrula* populations including *Q. quadrula apiculata* for the “apiculata‐East” population, *Q. quadrula quadrula* for the “quadrula” population, and *Q. quadrula forsheyi* (Lea 1859) for the “apiculata‐West” population. All four subspecies have been recognized previously (Frierson 1927; Haas 1969); however, low within‐drainage sample sizes outside the Mobile Basin prevent confident geographic delineation of these lineages.

### 4.2 River Drainages as Barriers and Conduits of Gene Flow

The strong yet clinal phylogeographic pattern within *Q. quadrula* (Figure 1) is best explained by allopatry followed by (re)colonization and secondary contact, punctuating the historical malleability of isolated river drainages as geographic barriers for aquatic taxa. The estimated timing of divergences (950–530 ka; Figure 4A) was during the mid‐late Pleistocene, a period that contained multiple ice ages with intervening warming periods (Hewitt 2011). Climatic fluctuations dramatically altered the river systems of eastern North America through pronounced sea level oscillations, glacial diversions, and deglaciation megafloods (Fildani et al. 2018). As recently as 20 ka, during the last glacial maximum, sea‐level dropped by approximately 121 m, exposing the continental shelf. Given the expansiveness of the shelf, certain Gulf Coast drainages would have extended another 100 km or more from their present‐day confluences (Donoghue 2011; Fairbanks 1989). Immediately following the last glacial maximum, during climatic warming, glacial runoff and flooding would have provided additional opportunities for stream capture, facilitating migration between presently isolated drainages before their divorce during sea‐level inundation (Burr and Page 1986; Marshall and Clarke 1999; Wickert 2016).

Molecular variation appears to reflect these geomorphological transfigurations. For all sampled characters, two major patterns emerged: (1) reduced variation at northern (Mississippi) range edges, and (2) two major clines, including a north–south cline extending from upstream Mississippi River populations (and Great Lakes for F‐mtDNA) south to southern Mississippi tributaries and a west–east cline surrounding the Gulf of Mexico from the Western Gulf into the Trinity & Sabine, southern Mississippi, Pearl & Pascagoula, and ending in the Mobile Basin. Reduced genetic variation seen in northern Mississippi drainage samples (Figures 1B–D and S10) is a major prediction of the rear‐leading edge hypothesis, which explains low genetic diversity at range edges as recent invasions into previously unoccupied habitat due to climate change (Hampe and Petit 2005; Hewitt 2004; Pironon et al. 2017; Stiller, Wilson, and Rouse 2023). As much of the northern occupied range (northern Mississippi Basin and Great Lakes) of *Q. quadrula* was uninhabitable during the last glacial maximum (Hewitt 2004; Lambeck and Chappell 2001), postglacial colonization logically explains the genetic composition of northern populations (Hoffman et al. 2017; Hoffman, Morris, and Zanatta 2018; Mathias et al. 2018; VanTassel et al. 2020). In contrast to northern populations, the geologic stability of the western range edge of *Q. quadrula* within the Western Gulf has led to phylogenetic differentiation that largely corresponds to isolated river drainages. Two clades within the Western Gulf region (Figures 2 and S6) differentiate a clade of predominately Rio Grande samples (southeastern range limit of *Q. quadrula*) from a clade containing samples from the Nueces and Navidad rivers. However, the Rio Grande clade also contains a sample from the Colorado River, a drainage northwest of the Grande, Nueces, and Navidad drainages. Populations from the Rio Grande are hypothesized to have been introduced based on its absence in fossil records, which may explain the close phylogenetic relationships between samples from the Rio Grande and a river basin several drainages northwest (Howells, Neck, and Murray 1996; Neck and Metcalf 1988).

Secondary contact of isolated populations explains molecular clines and mixed ancestry at drainage divides (e.g., Trinity & Sabine; Figure 1). These patterns could also be the result of incomplete lineage sorting, as we did find evidence of mito‐nuclear discordance (Figure S4), but the clear biogeographic pattern of all markers (Figure 1B–D) is consistent with introgression (Toews and Brelsford 2012). Despite the differing rates of evolution and effective population sizes of M‐mtDNA, F‐mtDNA, and nuDNA genomes, the lack of fixation of alleles within sympatric populations and shared sequences between neighboring river drainages suggest large population sizes, recent gene flow across contemporary geographic barriers, or both. Introgressive hybridization was particularly evident in the Mobile Basin, where we identified two phenotypes (*Q. apiculata* and *Q. rumphiana*) and individuals with either high “rumphiana” or mixed “rumphiana”‐“apiculata‐east” ancestry (Figure 3A). Individuals with high “apiculata‐east” (> 40%) ancestry were unanimously identified as *Q. apiculata* (Figure 2). However, these individuals were found to all be advanced (second generation) hybrids and possessed a hybrid index closer to parental “rumphiana” samples than to “apiculata‐east” parental samples from the neighboring Pascagoula drainage, indicating that hybridization is common and without fitness consequences (Figure 3B). This contact zone was located exclusively in the Coastal Plain and downstream from the Fall Line, the demarcation between coastal lowland and upland habitats (Figure 3A). Due to the steep elevational gradients, streams crossing the Fall Line often contain waterfalls and may have presented an upstream immigration barrier to the “apiculata‐east” lineage (Williams, Bogan, and Garner 2008). Further, the impoundment of the Alabama and Tombigbee rivers beginning in the 1800s may have facilitated the spread of the “apiculata‐east” lineage in the Mobile Basin. Impoundments have been shown to increase the dispersal of non‐native fishes, and in some cases, result in introgressive hybridization between endemic and invasive species (Bangs et al. 2018; Taylor, Knouft, and Hiland 2001). Catfishes, which serve as the host for *Q. quadrula* dispersal and metamorphosis, are common in anthropogenically modified habitats like impoundments (Michaletz and Dillard 1999; Michaletz and Sullivan 2002). Moreover, they are highly mobile fishes, which may have increased contact within the Mobile Basin but also across‐drainage dispersal when concurrently isolated drainages coalesced during low sea‐level events. Dispersal rates of freshwater mussels have unsurprisingly been shown to be correlated with the dispersal ability of their fish hosts (Zanatta and Wilson 2011). *Quadrula quadrula* invades newly available habitats quickly (Hoffman, Morris, and Zanatta 2018; Parmalee and Bogan 1998; VanTassel et al. 2020), and its phylogeographic congruence with its host fishes corroborates historical host‐mediated dispersal, but this is an area for further investigation (Padhi 2013, 2014).

### 4.3 Conclusions

Here, we emphasize spatial gradients of molecular variation and frequent hybridization in contact zones as a guide to infer the species boundaries and demographic history of a wide‐ranging freshwater mussel. For all sampled characters (F‐mtDNA, M‐mtDNA, nuDNA, and morphology), variation was clinal with respect to geography (Figure 1), with a notable contact zone in the Mobile Basin where admixed individuals collected from multiple localities were all identified as advanced (F2) or backcrossed hybrids. Consequently, we recognize *Q. quadrula* as a single cohesive species that spans many contemporaneously isolated populations. The overall evolutionary history of *Q. quadrula* suggests Pleistocene glacial cycles enabled phenotypic and phylogenetic diversification within isolated river drainages, followed by dispersal and colonization that effectively obliterated lineage diversification, creating gradations of biological variation across its widespread distribution. Despite strong clinal variation across the range of *Q. quadrula*, the persistence of morphological variation and its concordance with phylogenetic lineages supports the subspecific recognition of *Q. quadrula rumphiana*, endemic to the Mobile Basin. Although subspecies can be contentious, adopting subspecific nomenclature over ESUs or population segments for invertebrates in the United States allows for their listing and legislative protection under the Endangered Species Act. Freshwater mollusks face one of the highest rates of imperilment among organismal groups in the United States and worldwide (Böhm et al. 2021; Ricciardi and Rasmussen 1999). Their conservation is critical for the stability of aquatic ecosystems and the maintenance of ecosystem services they provide (e.g., biofiltration, nutrient recycling; Vaughn 2018).

## Author Contributions

S.M.K., N.A.J., C.H.S., and A.M.S. developed research ideas. S.M.K., N.A.J., C.H.S., B.E.S., J.T.G., and C.R.R. collected data and contributed to study design. S.M.K. and N.A.J. curated and published data. S.M.K. conducted all analyses and wrote the manuscript. All authors contributed to manuscript revisions.

## Conflicts of Interest

The authors declare no conflicts of interest.

## Supporting information


Data S1.

## Data Availability

All data and scripts are archived at https://doi.org/10.5066/P1PCMUWB. In addition, sequence data are archived at NCBI. See BioProject PRJNA1167272 for 3RAD data and Supplementary Table 1 for GenBank accession numbers. Benefits generated: All samples were collected with proper collection permits in collaboration with state and federal agencies. Vouchered specimens were deposited in public natural history museums. The results and data will be shared broadly with the malacological community including the American Fisheries Society's Common and Scientific Names Committee, the Freshwater Mollusk Conservation Society's Common and Scientific Names Committee, and Integrated Taxonomic Information System.
