# Supplementary material for: Secondary contact erodes Pleistocene diversification in a wide‐ranging freshwater mussel (Quadrula)
Source: Mol Ecol. 2024 Nov 14;34(1):e17572. doi: 10.1111/mec.17572 (PMC11665498; doi:10.1111/mec.17572)
Supplement: Supplementary file 1 — Data S1. [file MEC-34-e17572-s001.pdf]

MOLECULAR ECOLOGY

Supplemental Information for:

Secondary contact erodes Pleistocene diversification in a wide-ranging freshwater mussel (*Quadrula*)

Sean M. Keogh[a,b*], Nathan A. Johnson[c], Chase H. Smith[d], Bernard E. Sietman[e], Jeffrey T. Garner[f], Charles R. Randklev[g], & Andrew M. Simons[b,h]

[a]Gantz Family Collections Center, Field Museum of Natural History, 1400 South DuSable Lake Shore Drive, Chicago, IL 60605, USA
[b]Bell Museum of Natural History, University of Minnesota, 100 Ecology Building, 1987 Upper Buford Circle, St. Paul, MN 55108, USA
[c]US Geological Survey, Wetland and Aquatic Research Center, 7920 NW 71st Street, Gainesville, FL 32653, USA
[d]Department of Integrative Biology, University of Texas, Austin, TX 78712, USA
[e]Minnesota Department of Natural Resources, Center for Aquatic Mollusk Programs, Lake City, MN 55041, USA
[f]Alabama Division of Wildlife and Freshwater Fisheries, 350 County Road 275, Florence, AL 35633, USA
[g]Texas A&M Natural Resources Institute and AgriLife Research Center, 17360 Coit Road, Dallas, TX 75252, USA
[h]Department of Fisheries, Wildlife, and Conservation Biology, University of Minnesota, 135B Skok Hall, 2003 Upper Buford Circle, St. Paul, MN 55108, USA
*Corresponding author. E-mail address: keogh026@umn.edu

Table of Contents:

**Supplementary Tables**	Page 2-10
**Supplementary Figures**	Page 11-22

MOLECULAR ECOLOGY

Supplementary Tables

Supplementary Table 1: Sample metadata including morphological identifications, GenBank accession numbers, BioSample accession numbers, locality, and museum voucher information. See the 'Quad_samples' spreadsheet in Data Availability for more thorough information.

Taxon	Tissue	Morph Label	Morph ID	COI-F	NDI	ITSI	COI-M	RAD	Locality	Latitude	Longitude	Museum	Catalog No
Q. quadrula	SK18144	rum22857	apiculata	PQ038624	PQ057617	PQ143134	PQ142658	SAMN43991619	Alabama River	32.392701	-87.004616	JFBM	22857
Q. quadrula rumphiana	SK18155	rum22858_1	apiculata	PQ038625	PQ057618	PQ143135	PQ142659	SAMN43991620	Bogue Chitto AL	32.37014	-87.31969	JFBM	22858.1
Q. quadrula rumphiana	SK18156	rum22858_2	rumphiana	PQ038626	PQ057619		PQ142660	SAMN43991621	Bogue Chitto AL	32.37014	-87.31969	JFBM	22858.2
Q. quadrula rumphiana	SK18157	rum22858_3	apiculata	PQ038627			PQ142661	SAMN43991622	Bogue Chitto AL	32.37014	-87.31969	JFBM	22858.3
Q. quadrula rumphiana	SK18158	rum22858_4	rumphiana	PQ038628		PQ143136			Bogue Chitto AL	32.37014	-87.31969	JFBM	22858.4
Q. quadrula rumphiana	SK18164	rum22862_1	rumphiana	PQ038629	PQ057620			SAMN43991623	Sipsey River	33.331581	-87.761735	JFBM	22862.1
Q. quadrula rumphiana	SK18165	rum22862_2	rumphiana	PQ038630	PQ057621		PQ142662	SAMN43991624	Sipsey River	33.331581	-87.761735	JFBM	22862.2
Q. quadrula rumphiana	SK18166	rum22862_3	rumphiana	PQ038631			PQ142663	SAMN43991625	Sipsey River	33.331581	-87.761735	JFBM	22862.3
Q. quadrula rumphiana	SK18167	rum22862_4	rumphiana	PQ038632		PQ143137		SAMN43991626	Sipsey River	33.331581	-87.761735	JFBM	22862.4
Q. quadrula rumphiana	SK18168	rum22862_5	rumphiana	PQ038633		PQ143138		SAMN43991627	Sipsey River	33.331581	-87.761735	JFBM	22862.5
Q. quadrula	SK18179	qua22865_1	quadrula	PQ038637	PQ057623		PQ142664	SAMN43991628	Osage River	38.231031	-92.558275	JFBM	22865.1
Q. quadrula	SK18180	qua22865_2	quadrula	PQ038638	PQ057624			SAMN43991629	Osage River	38.231031	-92.558275	JFBM	22865.2
Q. quadrula	SK18181	qua22865_3	quadrula	PQ038639	PQ057625			SAMN43991630	Osage River	38.231031	-92.558275	JFBM	22865.3
Q. quadrula	SK18182	qua22865_4	quadrula	PQ038640		PQ143139	PQ142665	SAMN43991631	Osage River	38.231031	-92.558275	JFBM	22865.4
Q. quadrula	SK18183	qua22865_5	quadrula	PQ038641			PQ142666	SAMN43991632	Osage River	38.231031	-92.558275	JFBM	22865.5
Q. quadrula	SK18184	qua22865_6	quadrula	PQ038642			PQ142667		Osage River	38.231031	-92.558275	JFBM	22865.6
Q. quadrula	SK19054	qua1460_7	quadrula	PQ038652	PQ057628			SAMN43991633	Bayou LaGrue	34.09942	-91.16562	ASUMZ	1460.7
Q. quadrula	SK19058	rum438891_58	apiculata	PQ038654	PQ057629	PQ143140	PQ142668	SAMN43991634	Alabama River	31.6139	-87.55	FLMNH	438891.1

Q. quadrula	SK19059	rum438891_59	apiculata	PQ038655	PQ057630	PQ143141		SAMN43991635	Alabama River	31.6139	-87.55	FLMNH	438891.2
Q. quadrula	SK19060	rum438891_60	apiculata		PQ057631	PQ143142		SAMN43991636	Alabama River	31.6139	-87.55	FLMNH	438891.3
Q. quadrula	SK19061	qua438891_61	apiculata		PQ057632			SAMN43991637	Alabama River	31.6139	-87.55	FLMNH	438891.4
Q. quadrula	SK19062	api438891_62	apiculata		PQ057633	PQ143143		SAMN43991638	Alabama River	31.6139	-87.55	FLMNH	438891.5
Q. quadrula	SK19063	api438891_63	apiculata	PQ038656	PQ057634	PQ143144		SAMN43991639	Alabama River	31.6139	-87.55	FLMNH	438891.6
Q. quadrula	SK19064	qua438891_64	apiculata		PQ057635	PQ143145		SAMN43991640	Alabama River	31.6139	-87.55	FLMNH	438891.7
Q. quadrula	SK19065	rum438891_65	apiculata	PQ038657	PQ057636	PQ143146		SAMN43991641	Alabama River	31.6139	-87.55	FLMNH	438891.8
Q. quadrula	SK19066	qua438891_66	apiculata	PQ038658	PQ057637	PQ143147		SAMN43991642	Alabama River	31.6139	-87.55	FLMNH	438891.9
Q. quadrula	SK19067	qua438891_67	apiculata	PQ038659	PQ057638	PQ143148		SAMN43991643	Alabama River	31.6139	-87.55	FLMNH	########
Q. quadrula	SK19068	rum438891_68	apiculata		PQ057639	PQ143149		SAMN43991644	Alabama River	31.6139	-87.55	FLMNH	438891.1
Q. quadrula	SK19069	qua438891_69	apiculata	PQ038660	PQ057640	PQ143150			Alabama River	31.6139	-87.55	FLMNH	438891.1
Q. quadrula	SK19070	qua438891_70	apiculata		PQ057641	PQ143151		SAMN43991645	Alabama River	31.6139	-87.55	FLMNH	438891.1
Q. quadrula rumphiana	SK19083	rum23034_1	rumphiana	PQ038661			PQ142669	SAMN43991646	Big Canoe Creek	33.848566	-86.250472	JFBM	23034.1
Q. quadrula rumphiana	SK19084	rum23034_2	rumphiana	PQ038662				SAMN43991647	Big Canoe Creek	33.848566	-86.250472	JFBM	23034.2
Q. quadrula rumphiana	SK19085	rum23034_3	rumphiana	PQ038663			PQ142670	SAMN43991648	Big Canoe Creek	33.848566	-86.250472	JFBM	23034.3
Q. quadrula rumphiana	SK19086/20049	api438826_1	apiculata	PQ038664	PQ057642	PQ143152		SAMN43991649	Bogue Chitto AL	32.31001	-87.27863	FLMNH	438826.1
Q. quadrula rumphiana	SK19087/20050	api438826_2	apiculata	PQ038665	PQ217097	PQ143153		SAMN43991650	Bogue Chitto AL	32.31001	-87.27863	FLMNH	438826.2
Q. quadrula rumphiana	SK20002	rum438826_3	apiculata	PQ038716				SAMN43991651	Bogue Chitto AL	32.31001	-87.27863	FLMNH	438826.3
Q. quadrula rumphiana	SK19090	rum438419_1	apiculata	PQ038666	PQ057643	PQ143154			Black Warrior	32.555	-87.84409	FLMNH	438419.1
Q. quadrula rumphiana	SK19091	rum438419_2	rumphiana	PQ038667	PQ057644	PQ143155		SAMN43991652	Black Warrior	32.555	-87.84409	FLMNH	438419.2
Q. quadrula	SK19092	api441004_1	apiculata	PQ038668	PQ057645	PQ143156		SAMN43991653	Navidad River	28.91412	-96.56886	FLMNH	441004.1
Q. quadrula	SK19093	api441004_2	apiculata	PQ038669	PQ057646	PQ143157		SAMN43991654	Navidad River	28.91412	-96.56886	FLMNH	441004.2
Q. quadrula	SK19094	qua438561_1	apiculata	PQ038670	PQ057647	PQ143158	PQ142671	SAMN43991655	Nueces River	28.47405	-98.25166	FLMNH	438561.1
Q. quadrula	SK19095	qua438561_2	apiculata	PQ038671	PQ057648	PQ143159		SAMN43991656	Nueces River	28.47405	-98.25166	FLMNH	438561.2
Q. quadrula	QapiNue088	qua438561_3	apiculata	PQ038598	PQ057591	PQ143118	PQ142656		Nueces River	28.47405	-98.25166	FLMNH	438561.3
Q. quadrula	QapiNue089	api438561_4	apiculata	PQ038599	PQ057592	PQ143119			Nueces River	28.47405	-98.25166	FLMNH	438561.4

Q. quadrula	QapiNue090	api438561_5	apiculata	PQ038600	PQ057593	PQ143120	PQ142657		Nueces River	28.47405	-98.25166	FLMNH	438561.5
Q. quadrula	SK19096	api438801_1	apiculata	PQ038672	PQ057649	PQ143160		SAMN43991657	Nueces River	28.382964	-98.109617	FLMNH	438801.1
Q. quadrula	SK19097	api438804_1	apiculata	PQ038673	PQ057650	PQ143161		SAMN43991658	Nueces River	28.337458	-98.086636	FLMNH	438804.1
Q. quadrula	SK19098			PQ038674	PQ057651				Red River KY	37.84167	-83.76	UA	3609
Q. quadrula	SK19099	QapiPrl125	apiculata	PQ038675	PQ057652	PQ143162		SAMN43991659	Bogue Chitto LA	30.62254	-89.87655	FLMNH	439143
Q. quadrula	QapiPrl126	QapiPrl126	apiculata	PQ038601	PQ057594	PQ143121			Bogue Chitto LA	30.62254	-89.87655	FLMNH	439143
Q. quadrula	SK19100	QquaOhi012	quadrula	PQ038676	PQ057653	PQ143163		SAMN43991660	Ohio River	39.39123122	-81.42094424	FLMNH	439156
Q. quadrula	SK19101	QquaOhi013	quadrula	PQ038677	PQ057654	PQ143164		SAMN43991661	Ohio River	39.39123122	-81.42094424	FLMNH	439156
Q. quadrula	SK19102	QquaOhi015	quadrula	PQ038678	PQ057655	PQ143165			Ohio River	39.39123122	-81.42094424	FLMNH	439156
Q. quadrula	QquaOhi016	QquaOhi016	quadrula	PQ038614	PQ057607				Ohio River	39.39123122	-81.42094424	FLMNH	439156
Q. quadrula	SK19104			PQ038680	PQ057657				Ouachita River	32.95613	-92.07864	ASUMZ	QF4
Q. quadrula	SK19109	qua23040_1	quadrula	PQ038682				SAMN43991662	Iowa River	41.555722	-91.519682	JFBM	23040.1
Q. quadrula	SK19110	qua23040_2	quadrula	PQ038683				SAMN43991663	Iowa River	41.555722	-91.519682	JFBM	23040.2
Q. quadrula	SK19111	qua23040_3	quadrula	PQ038684				SAMN43991664	Iowa River	41.555722	-91.519682	JFBM	23040.3
Q. quadrula	SK19112	qua23040_4	quadrula	PQ038685			PQ142672		Iowa River	41.555722	-91.519682	JFBM	23040.4
Q. quadrula	SK19113	qua23040_5	quadrula	PQ038686			PQ142673		Iowa River	41.555722	-91.519682	JFBM	23040.5
Q. quadrula	SK19114	qua23040_6	quadrula	PQ038687					Iowa River	41.555722	-91.519682	JFBM	23040.6
Q. quadrula	SK19115	qua23040_7	quadrula	PQ038688					Iowa River	41.555722	-91.519682	JFBM	23040.7
Q. quadrula	SK19116	qua23040_8	quadrula	PQ038689					Iowa River	41.555722	-91.519682	JFBM	23040.8
Q. quadrula	SK19117	qua23041	quadrula	PQ038690	PQ057658		PQ142674		Mississippi River	43.634071	-91.241814	JFBM	23041
Q. quadrula	SK19118	qua23042	quadrula	PQ038691	PQ057659				Mississippi River	43.603198	-91.230798	JFBM	23042
Q. quadrula	SK19119	qua23043_1	quadrula	PQ038692			PQ142675	SAMN43991665	Mississippi River	43.664114	-91.235728	JFBM	23043.1
Q. quadrula	SK19120	qua23043_2	quadrula	PQ038693			PQ142676	SAMN43991666	Mississippi River	43.664114	-91.235728	JFBM	23043.2
Q. quadrula	SK19121	qua23044_1	quadrula	PQ038694			PQ142677		Mississippi River	42.056605	-90.14924	JFBM	23044.1
Q. quadrula	SK19122	qua23044_2	quadrula	PQ038695	PQ057660				Mississippi River	42.056605	-90.14924	JFBM	23044.2
Q. quadrula	SK19123	qua23044_3	quadrula	PQ038696	PQ057661		PQ142678		Mississippi River	42.056605	-90.14924	JFBM	23044.3
Q. quadrula	SK19124	qua23044_4	quadrula	PQ038697	PQ057662				Mississippi River	42.056605	-90.14924	JFBM	23044.4
Q. quadrula	SK19125	qua23044_5	quadrula	PQ038698	PQ057663		PQ142679		Mississippi River	42.056605	-90.14924	JFBM	23044.5
Q. quadrula	SK19126	qua23045_1	quadrula	PQ038699	PQ057664			SAMN43991667	Mississippi River	45.030727	-93.282595	JFBM	23045.1

Q. quadrula	SK19127	qua23045_2	quadrula	PQ038700	PQ057665			SAMN43991668	Mississippi River	45.030727	-93.282595	JFBM	23045.2
Q. quadrula	SK19128	qua23045_3	quadrula	PQ038701	PQ057666		PQ142680		Mississippi River	45.030727	-93.282595	JFBM	23045.3
Q. quadrula	SK19163	qua8378_2	apiculata	PQ038702				SAMN43991669	Trinity River	33.305606	-96.607848	TXA&M-NRI	8378.2
Q. quadrula	SK19164	qua8378_3	apiculata	PQ038703	PQ057667				Trinity River	33.305606	-96.607848	TXA&M-NRI	8378.3
Q. quadrula	SK19165	qua8584_1	quadrula	PQ038704				SAMN43991670	Red River	33.686069	-94.994325	TXA&M-NRI	8584.1
Q. quadrula	SK19166	qua8584_2	quadrula	PQ038705	PQ057668				Red River	33.686069	-94.994325	TXA&M-NRI	8584.2
Q. quadrula	SK19167	qua8584_3	quadrula	PQ038706					Red River	33.686069	-94.994325	TXA&M-NRI	8584.3
Q. quadrula	SK19168	qua8602_1	quadrula	PQ038707				SAMN43991671	Red River	33.654324	-96.048929	TXA&M-NRI	8602.1
Q. quadrula	SK19169	qua8602_2	quadrula	PQ038708					Red River	33.654324	-96.048929	TXA&M-NRI	8602.2
Q. quadrula	SK19170	qua8602_3	quadrula	PQ038709					Red River	33.654324	-96.048929	TXA&M-NRI	8602.3
Q. quadrula	SK19171	qua8610_1	ambigious	PQ038710					Red River	33.657660	-96.055550	TXA&M-NRI	8610.1
Q. quadrula	SK19172	qua8610_2	quadrula	PQ038711					Red River	33.657660	-96.055550	TXA&M-NRI	8610.2
Q. quadrula	SK19173	qua8656_1	quadrula	PQ038712	PQ057669				Red River	33.826894	-95.412156	TXA&M-NRI	8656.1
Q. quadrula	SK19174	qua8656_2	ambigious		PQ057670			SAMN43991672	Red River	33.826894	-95.412156	TXA&M-NRI	8656.2
Q. quadrula	SK19175	qua8656_3	ambigious		PQ057671				Red River	33.826894	-95.412156	TXA&M-NRI	8656.3
Q. quadrula	SK19176	qua8566_1	ambigious	PQ038713	PQ057672				Little River	33.976200	-94.932200	TXA&M-NRI	8566.1
Q. quadrula	SK19177	qua8566_2	ambigious	PQ038714					Little River	33.976200	-94.932200	TXA&M-NRI	8566.2
Q. quadrula	SK19178	qua8566_3	quadrula	PQ038715					Little River	33.976200	-94.932200	TXA&M-NRI	8566.3
Q. quadrula	8262	api8262	apiculata	PQ038583	PQ057573	PQ143107		SAMN43991673	Trinity River	31.6632089	-95.79061759	TXA&M-NRI	8262
Q. quadrula	8382.1	api8382_1	apiculata	PQ038584	PQ057574				Rio Grande	27.602238	-99.580507	TXA&M-NRI	8382.1
Q. quadrula	8382.2	api8382_2	apiculata	PQ038585					Rio Grande	27.602238	-99.580507	TXA&M-NRI	8382.2
Q. quadrula	8427.1	qua8427_1	apiculata	PQ038586	PQ057575	PQ143108			Brazos River	29.78500	-95.74700	TXA&M-NRI	8427.1
Q. quadrula	8427.2	api8427_2	apiculata	PQ038587	PQ057576	PQ143109			Brazos River	29.785	-95.747	TXA&M-NRI	8427.2
Q. quadrula	8437.1			PQ038588				SAMN43991674	Colorado River	30.90116	-99.91932	TXA&M-NRI	8437.1
Q. quadrula	8437.2			PQ038589	PQ057577				Colorado River	30.90116	-99.91932	TXA&M-NRI	8437.2
Q. quadrula	8470.2	api8470_2	apiculata	PQ038590	PQ057578	PQ143110			Trinity River	30.88456	-95.77792	TXA&M-NRI	8470.2
Q. quadrula	8122.1	qua8122_1	quadrula	PQ038581	PQ057572	PQ143106			Red River	33.82702	-95.41176	TXA&M-NRI	8122.1
Q. quadrula	8122.2	qua8122_2	quadrula	PQ038582					Red River	33.82702	-95.41176	TXA&M-NRI	8122.2
Q. quadrula	SK20006	qua440990_1	apiculata					SAMN43991675	Galveston Bay	29.59798	-95.28663	FLMNH	440990.1

Q. quadrula	SK20007	qua440990_2	apiculata	PQ038717	PQ057673			SAMN43991676	Galveston Bay	29.59798	-95.28663	FLMNH	440990.2
Q. quadrula	SK20010/041	qua438313_1	apiculata	PQ038718	PQ057674	PQ143166		SAMN43991677	Trinity River	30.627278	-95.008917	FLMNH	438313.1
Q. quadrula	SK20015/046	qua438313_6	apiculata	PQ038719			PQ142681	SAMN43991678	Trinity River	30.627278	-95.008917	FLMNH	438313.6
Q. quadrula	SK20021	api438809_1	apiculata	PQ038721			PQ142682		Rio Grande	27.62609	-99.58903	FLMNH	438809.1
Q. quadrula	SK20022	api438809_7	apiculata	PQ038722			PQ142683	SAMN43991679	Rio Grande	27.62609	-99.58903	FLMNH	438809.7
Q. quadrula	SK20023	api438809_2	apiculata	PQ038723					Rio Grande	27.62609	-99.58903	FLMNH	438809.2
Q. quadrula	SK20024	api438809_3	apiculata	PQ038724			PQ142684	SAMN43991680	Rio Grande	27.62609	-99.58903	FLMNH	438809.3
Q. quadrula	SK20025	api438809_4	apiculata	PQ038725				SAMN43991681	Rio Grande	27.62609	-99.58903	FLMNH	438809.4
Q. quadrula	SK20026	api438809_5	apiculata	PQ038726					Rio Grande	27.62609	-99.58903	FLMNH	438809.5
Q. quadrula	SK20027	api438809_6	apiculata	PQ038727					Rio Grande	27.62609	-99.58903	FLMNH	438809.6
Q. quadrula	SK20028	api438809_8	apiculata	PQ038728					Rio Grande	27.62609	-99.58903	FLMNH	438809.8
Q. quadrula	SK20029	api438809_9	apiculata	PQ038729			PQ142685		Rio Grande	27.62609	-99.58903	FLMNH	438809.9
Q. quadrula	SK20030	api438809_10	apiculata	PQ038730				SAMN43991682	Rio Grande	27.62609	-99.58903	FLMNH	438809.1
Q. quadrula	SK20035	QapiNec156	apiculata	PQ038731	PQ057676	PQ143167			Neches River	30.659012	-94.170894	FLMNH	439196
Q. quadrula	SK20036	Qapi007Pas	apiculata				PQ142686	SAMN43991683	Pascagoula River	30.63228	-88.652401	FLMNH	438884.1
Q. quadrula	SK20038	qua438884_3	apiculata	PQ038732	PQ057677	PQ143168		SAMN43991684	Pascagoula River	30.63228	-88.652401	FLMNH	438884.3
Q. quadrula	SK20039	qua438884_4	apiculata	PQ038733	PQ057678	PQ143169		SAMN43991685	Pascagoula River	30.63228	-88.652401	FLMNH	438884.4
Q. quadrula rumphiana	SK21001	rum23111_1	rumphiana	PQ038734	PQ057679				Black Warrior River	33.736553	-86.928699	JFBM	23111.1
Q. quadrula	SK21002	rum23111_2	apiculata	PQ038735	PQ057680				Black Warrior River	33.736553	-86.928699	JFBM	23111.2
Q. quadrula	SK21003	rum23111_3	apiculata	PQ038736	PQ057681				Black Warrior River	33.736553	-86.928699	JFBM	23111.3
Q. quadrula	ASU1234.1	qua1234_1	quadrula		PQ057579				Arkansas River	35.11874	-92.5532	ASUMZ	1234.1
Q. quadrula	ASU1234.2	qua1234_2	quadrula		PQ057581				Arkansas River	35.11874	-92.5532	ASUMZ	1234.2
Q. quadrula	ASU1234.3	qua1234_3	quadrula	PQ038591	PQ057582	PQ143111			Arkansas River	35.11874	-92.5532	ASUMZ	1234.3
Q. quadrula	ASU1234.4	qua1234_4	quadrula		PQ057583				Arkansas River	35.11874	-92.5532	ASUMZ	1234.4
Q. quadrula	ASU1234.9	qua1234_9	quadrula		PQ057584				Arkansas River	35.11874	-92.5532	ASUMZ	1234.9
Q. quadrula	ASU1234.10	qua1234_10	quadrula		PQ057580				Arkansas River	35.11874	-92.5532	ASUMZ	1234.10
Q. quadrula	SK21111	SK21111	quadrula	PQ038746					St. Croix River	45.395003	-92.664611		
Q. quadrula	SK21112	SK21112	quadrula	PQ038747					St. Croix River	45.395003	-92.664611		

Q. quadrula	SK21115	SK21115	apiculata	PQ038748			Little River	33.936448	-94.829126		
Q. quadrula	SK21119	SK21119	apiculata	PQ038749			Little River	33.936448	-94.829126		
Q. quadrula	SK21120	SK21120	quadrula	PQ038750			Little River	33.936448	-94.829126		
Q. quadrula	SK21123	SK21123	ambigious	PQ038751			Little River	33.936448	-94.829126		
Q. quadrula	SK21125	SK21125	quadrula	PQ038752			Little River	33.936448	-94.829126		
Q. quadrula	SK21126	SK21126	quadrula	PQ038753			Little River	33.936448	-94.829126		
Q. quadrula	SK21127	SK21127	quadrula	PQ038754			Little River	33.936448	-94.829126		
Q. quadrula	152	qua23178	quadrula	PQ038578			Glover River	34.032063	-94.9393	JFBM	23178
Q. quadrula	178	qua23179_1	apiculata	PQ038579			Kiamichi River	34.656876	-95.055331	JFBM	23179.1
Q. quadrula	179	qua23179_2	apiculata	PQ038580			Kiamichi River	34.656876	-95.055331	JFBM	23179.2
Q. quadrula	QapiGal016	QapiGal016	apiculata	PQ038594	PQ057587	PQ143114	Galveston Bay	29.789741	-95.623742	FLMNH	438818
Q. quadrula	QapiGal017	QapiGal017	apiculata	PQ038595	PQ057588	PQ143115	Galveston Bay	29.789741	-95.623742	FLMNH	438818
Q. quadrula	QapiNav028	QapiNav028	apiculata	PQ038596	PQ057589	PQ143116	Navidad River	28.95756	-96.54243	FLMNH	438814
Q. quadrula	QapiNav029	QapiNav029	apiculata	PQ038597	PQ057590	PQ143117	Navidad River	28.95756	-96.54243	FLMNH	438814
Q. quadrula	QapiCol044	QapiCol044	apiculata	PQ038592	PQ057585	PQ143112	San Saba River	31.20738	-98.68761	FLMNH	441088
Q. quadrula	QapiTri063	qua441182_1	apiculata	PQ038607	PQ057600	PQ143126	Trinity River	32.592543	-96.486377	FLMNH	441182.1
Q. quadrula	QapiTri064	api441182_2	apiculata	PQ038608	PQ057601	PQ143127	Trinity River	32.592543	-96.486377	FLMNH	441182.2
Q. quadrula	QapiCol065	api441183	apiculata	PQ038593	PQ057586	PQ143113	Colorado River	31.4831003	-99.030595	FLMNH	441183
Q. quadrula	QapiRGr081	api438554_2	apiculata	PQ038603	PQ057596	PQ143122	Rio Grande	27.63774	-99.62695	FLMNH	438554.2
Q. quadrula	QapiRGr082	api438554_3	apiculata	PQ038604	PQ057597	PQ143123	Rio Grande	27.63774	-99.62695	FLMNH	438554.3
Q. quadrula	QapiRGr083	api438554_4	apiculata	PQ038605	PQ057598	PQ143124	Rio Grande	27.63774	-99.62695	FLMNH	438554.4
Q. quadrula	QapiRGr092	QapiRGr092	apiculata	PQ038606	PQ057599	PQ143125	Rio Grande	27.64193	-99.60222	FLMNH	438747
Q. quadrula	QquaRed002	QquaRed002	quadrula	PQ038615	PQ057608	PQ143130	Red River	33.854656	-95.341305	FLMNH	438787
Q. quadrula	QapiTri107	api438951_1	quadrula	PQ038609	PQ057602	PQ143128	Trinity River	33.036203	-97.1759	FLMNH	438951.1
Q. quadrula	QapiTri113	api438952_2	apiculata	PQ038610	PQ057603	PQ143129	Trinity River	32.980521	-97.09841	FLMNH	438952.2
Q. quadrula	QrumMob003			PQ038616	PQ057609		Sipsey River	33.1025	-87.95	UA	1489
Q. quadrula	QspeMob015			PQ038620	PQ057613		Alabama River	32.31	-87.28	UA	1726
Q. quadrula	QapiRed139			PQ038602	PQ057595		Little River	33.691899	-93.965148	ASUMZ	1230.7
Q. quadrula	QrumTom005	QrumTom005	rumphiana	PQ038617	PQ057610	PQ143131	Sipsey River	33.040556	-88.113611	FLMNH	439169

Q. quadrula	QrumTom008	QrumTom008	rumphiana	PQ038618	PQ057611	PQ143132			Sipsey River	33.040556	-88.113611	FLMNH	439169
Q. quadrula	QrumTom011	QrumTom011	rumphiana	PQ038619	PQ057612	PQ143133			Sipsey River	33.040556	-88.113611	FLMNH	439169
Q. quadrula	QfraOua017			PQ038611	PQ057604				Ouachita River	33.03438	-92.09618	ASUMZ	QF1
Q. quadrula	QfraOua019			PQ038612	PQ057605				Ouachita River	32.95613	-92.07864	ASUMZ	QF3
Q. quadrula	QfraOua021			PQ038613	PQ057606				Ouachita River	32.95613	-92.07864	ASUMZ	QF5
C. tuberculata	SK20018			PQ038720	PQ057675			SAMN43991686	Nolichucky River	36.1451307	-83.1525792	FLMNH	438245
Q. verrucosa	SK19103			PQ038679	PQ057656			SAMN43991687	Sipsey River	33.121505	-87.911094	FLMNH	439171
Q. verrucosa	SK19108			PQ038681				SAMN43991688	St. Croix River	45.394149	-92.663579	JFBM	23039
Q. verrucosa	QverCol013			PQ038621	PQ057614				Llano River	30.658673	-99.3245	FLMNH	438746
Q. nobilis	SK18113			PQ038622	PQ057615			SAMN43991689	Tennessee River	34.734213	-87.773591	JFBM	22855.2
Q. nobilis	SK18141			PQ038623	PQ057616				Alabama River	32.392701	-87.004616	JFBM	22856.4
Q. nobilis	SK18169			PQ038634	PQ057622			SAMN43991690	Osage River	38.231031	-92.558275	JFBM	22864.1
Q. nobilis	SK18177			PQ038635	PQ217094		PQ202238		Osage River	38.231031	-92.558275	JFBM	22864.9
Q. nobilis	SK18178			PQ038636	PQ217095		PQ202239		Osage River	38.231031	-92.558275	JFBM	22864.10.
Q. nobilis	SK19055			PQ038653	PQ217096			SAMN43991691	Bayou LaGrue	34.09942	-91.16562	ASUMZ	1460.8
Q. nobilis	SK86191			PQ038755	PQ057691				Red River	33.87305	-95.5412	TXA&M-NRI	8619.1
Q. fragosa	SK18188			PQ038643				SAMN43991692	St. Croix River	45.394566	-92.663184	uncat-swab	
Q. fragosa	SK18189			PQ038644	PQ057626			SAMN43991693	St. Croix River	45.394566	-92.663184	uncat-swab	
Q. fragosa	SK-18-190			PQ038645	PQ057627			SAMN43991694	St. Croix River	45.394566	-92.663184	uncat-swab	
Q. fragosa	SK18191			PQ038646				SAMN43991695	St. Croix River	45.394566	-92.663184	uncat-swab	
Q. fragosa	SK18192			PQ038647					St. Croix River	45.394566	-92.663184	uncat-swab	
Q. fragosa	SK18193			PQ038648					St. Croix River	45.394566	-92.663184	uncat-swab	
Q. fragosa	SK18194			PQ038649					St. Croix River	45.394566	-92.663184	uncat-swab	
Q. fragosa	SK18195			PQ038650					St. Croix River	45.394566	-92.663184	uncat-swab	
Q. fragosa	SK18196			PQ038651					St. Croix River	45.394566	-92.663184	uncat-swab	
Q. fragosa	SK21004			PQ038737	PQ057682				Little River	33.936362	-94.830001	uncat-swab	
Q. fragosa	SK21005			PQ038738	PQ057683				Little River	33.936362	-94.830001	uncat-swab	
Q. fragosa	SK21006			PQ038739	PQ057684				Little River	33.936362	-94.830001	uncat-swab	
Q. fragosa	SK21008			PQ038740	PQ057685				Little River	33.936362	-94.830001	uncat-swab	

Q. fragosa	SK21009	PQ038741	PQ057686	Little River	33.936362	-94.830001	uncat-swab
Q. fragosa	SK21010	PQ038742	PQ057687	Little River	33.936362	-94.830001	uncat-swab
Q. fragosa	SK21011	PQ038743	PQ057688	Little River	33.936362	-94.830001	uncat-swab
Q. fragosa	SK21012	PQ038744	PQ057689	Little River	33.936362	-94.830001	uncat-swab
Q. fragosa	SK21013	PQ038745	PQ057690	Little River	33.936362	-94.830001	uncat-swab

Supplementary Table 2: 3RAD datasets and files used in analyses including substitution models used for IQ-TREE analyses. Some ipyrad files were further manipulated as explained in the methods.

Analysis	ipyrad Assembly	Minimum Samples/Locus	ipyrad File	IQ-TREE Model
IQ-TREE	"all"	7	.phy	GTR+F+R2
IQ-TREE	"all"	17	.phy	GTR+F+R7
IQ-TREE	"all"	34	.phy	TVM+F+R5
IQ-TREE	"all"	51	.phy	K3Pu+F+R2
IQ-TREE	"nomob"	17	.phy	GTR+F+R2
sNMF	"ingroup"	51	.ugeno	-
STRUCTURE	"ingroup"	51	.snps.hdf5	-
Isolation by distance	"ingroup"	34	.vcf	-
PCA	"ingroup"	34	.snps.hdf5	-
SNAPP	"all"	34	.usnps	-
gghybrid	"ingroup"	34	.ustr	-
newhybrids	"ingroup"	34	.vcf	-
delimitR	"ingroup"	34	.vcf	-

Supplementary Figures

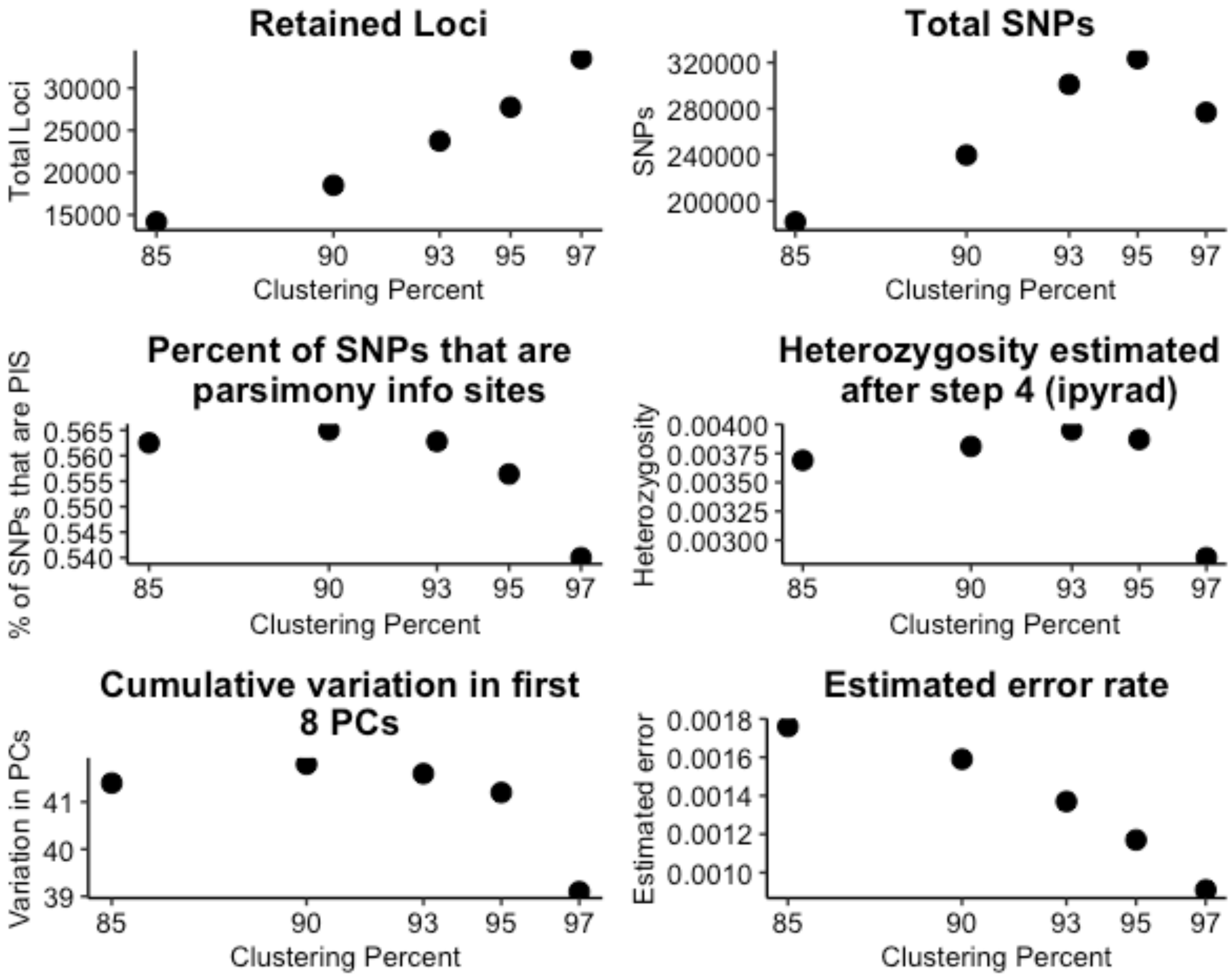

Supplementary Figure 1: Assessing the impact of clustering threshold on number of retained loci, total SNPs, parsimony informative SNPs, heterozygosity, variation explained by first 8 principal components, and estimated error rate.

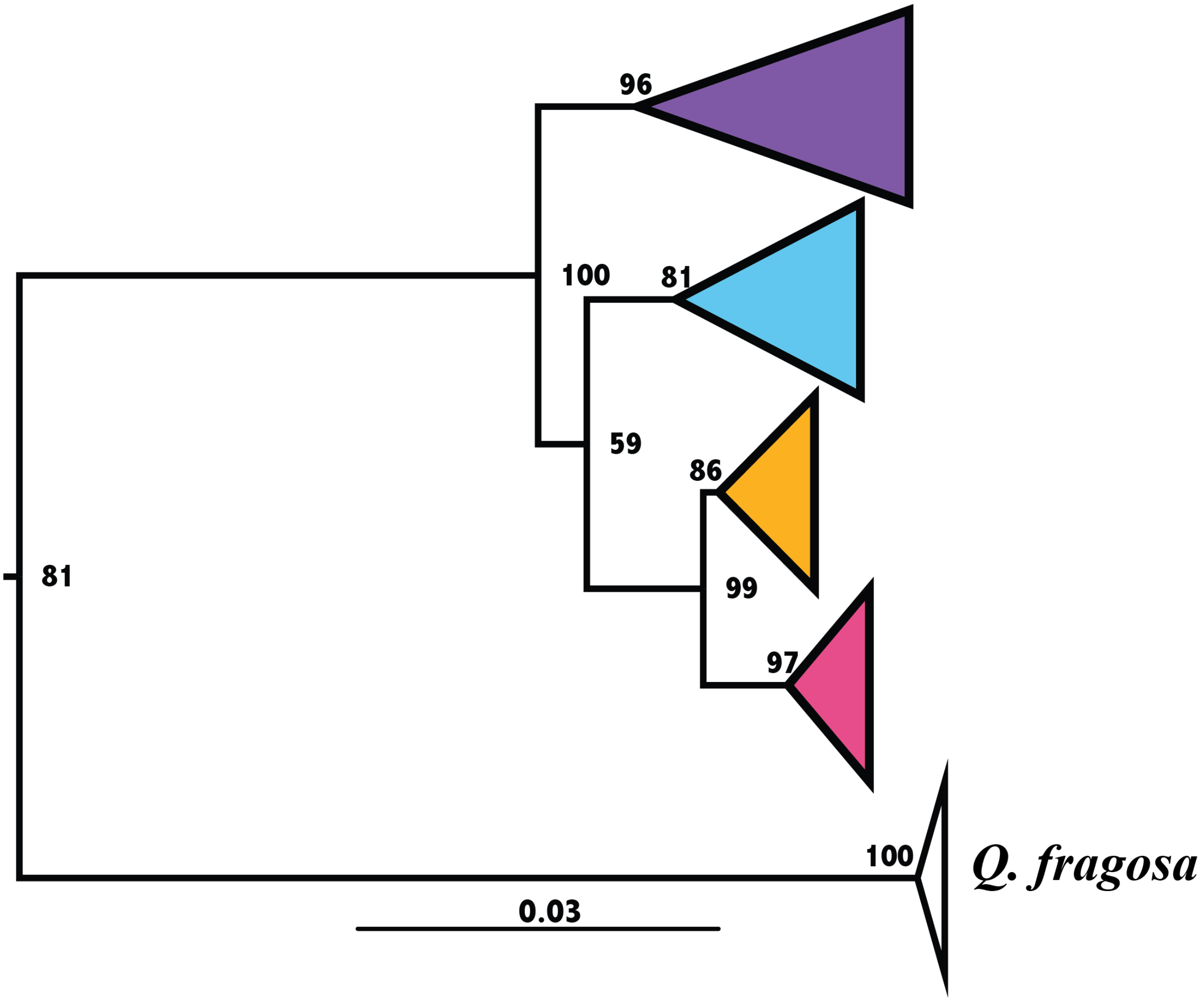

Supplementary Figure 2: The best tree using F-mtDNA (COI & NDI) produced from RAxML with bootstrap support values at informative nodes. Color scheme is identical to Figures 1-2. Outgroups were pruned from tree visualization. The phylogeny was rooted at the midpoint.

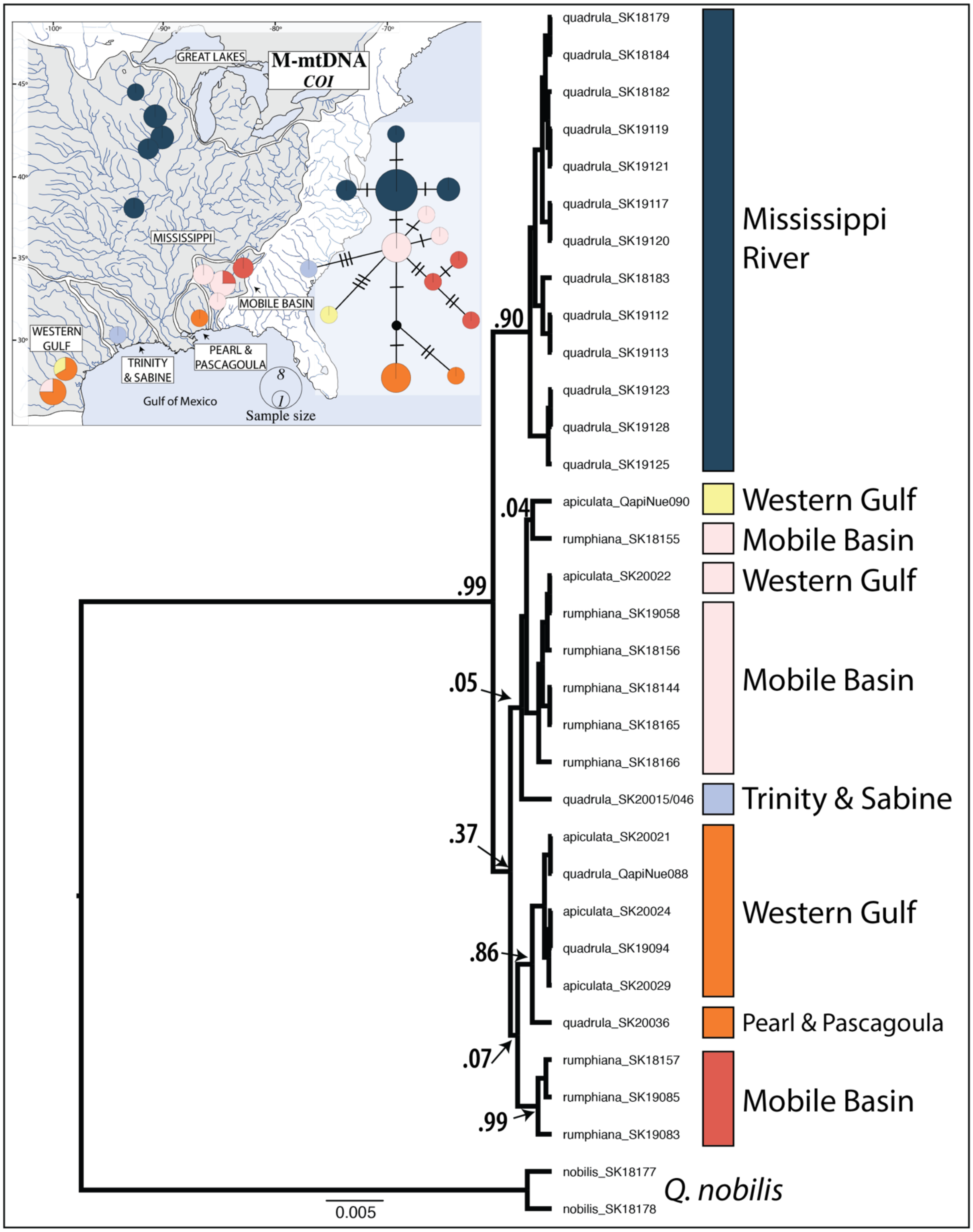

Supplementary Figure 3: Estimated phylogeny using M-mtDNA (COI-M) of *Q. quadrula* samples. Colors correspond to TCS haplotype network groups. Figure 1C reproduced to visualize spatial sampling.

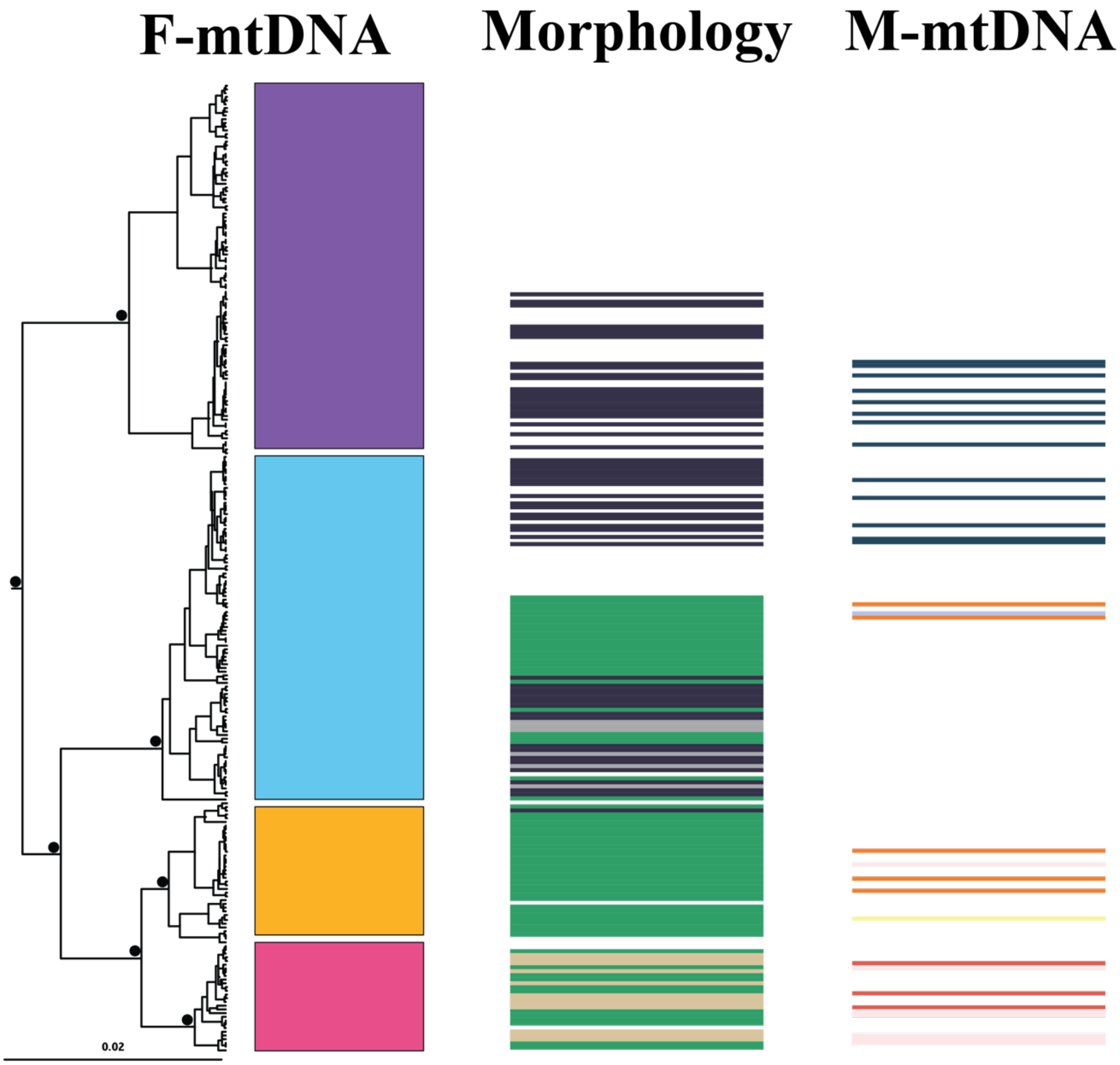

Supplementary Figure 4: Congruence of F-mtDNA phylogeny (from BEAST2) with morphological identification and M-mtDNA haplotypes. White space denotes samples that were not sampled for morphology and/or M-mtDNA and gray color in the morphology column shows specimens with ambiguous shell morphology (i.e. lack of consensus). Color schemes are consistent with Figure 1.

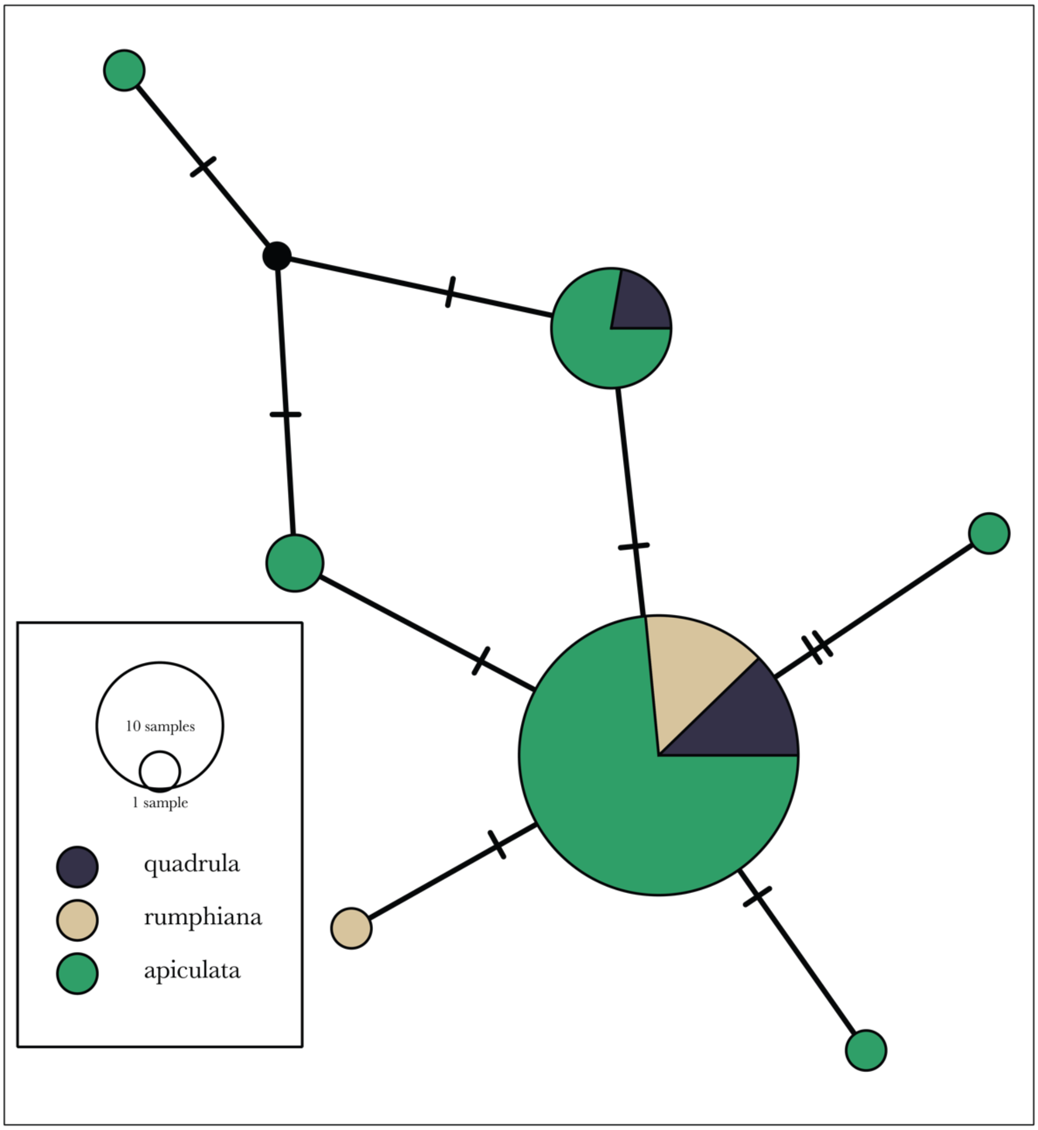

Supplementary Figure 5: TCS haplotype network of ITSI. Samples colored by consensus morphological identification.

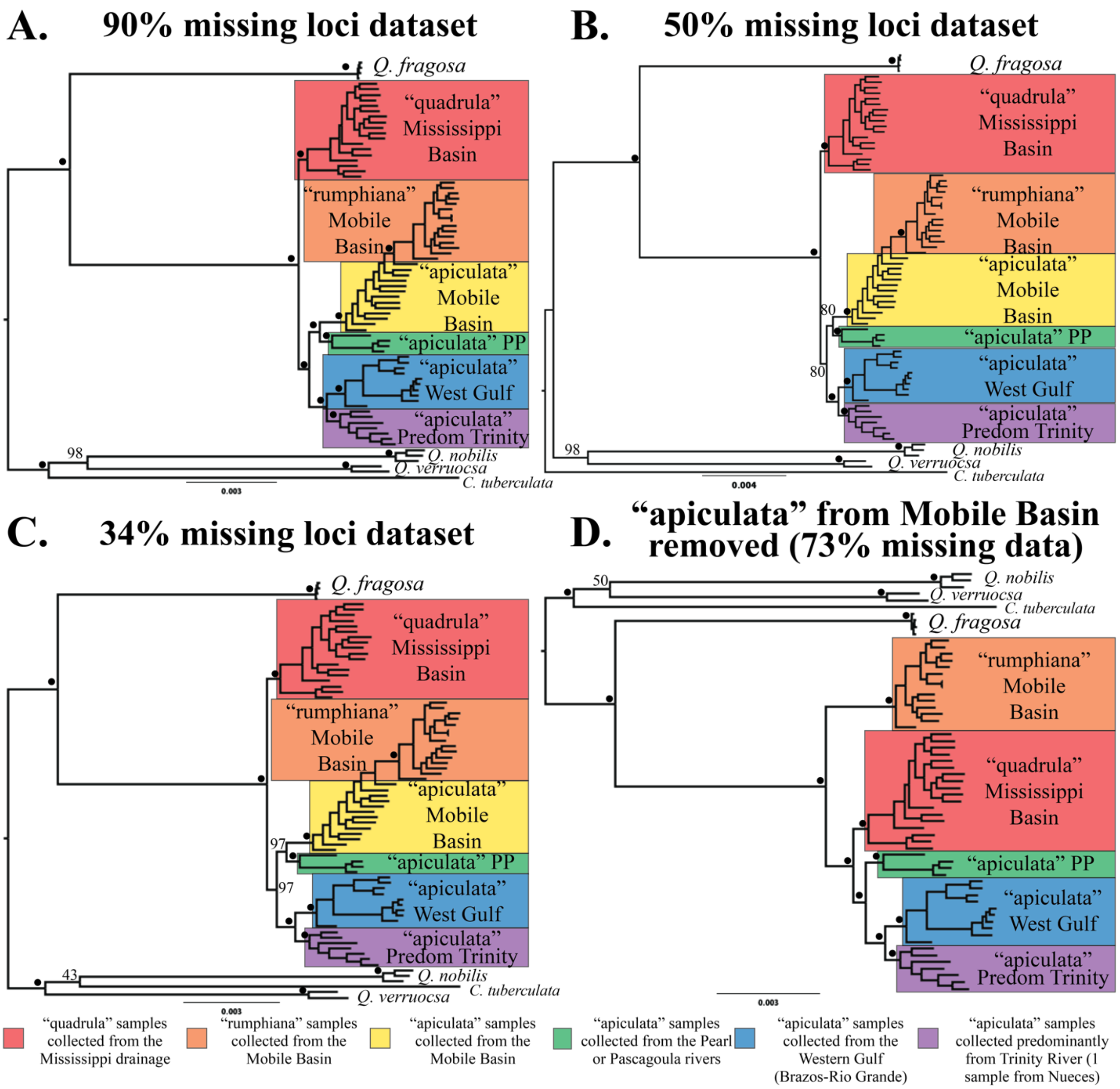

Supplementary Figure 6: IQ-TREE phylogenies constructed from 'msl7' (A.), 'msl34' (B.), 'msl51' (C.), and 'nomob' (D.) SNPs datasets. Labels at terminal branches show the majority population from sNMF analysis and geography of where specimens were collected. Black circles at nodes indicate 100 bootstrap support. Refer to Supplementary Table 1 for substitution models and minimum samples per locus.

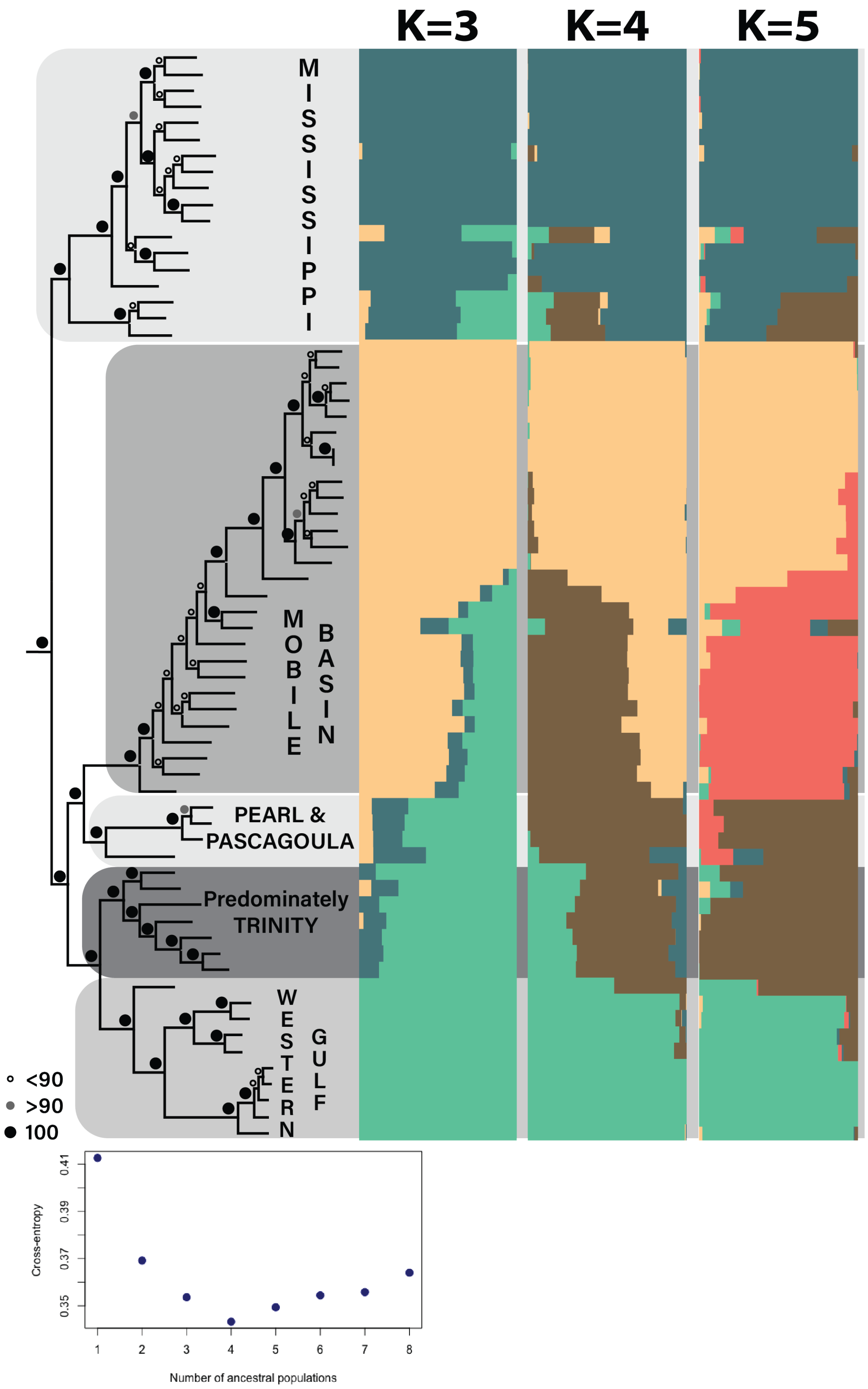

Supplementary Figure 7: IQ-TREE phylogeny (same as Figure 2) with sNMF ancestry coefficients for K=3-5 and cross-entropy scores for K=1-8 using SNPs (nuDNA).

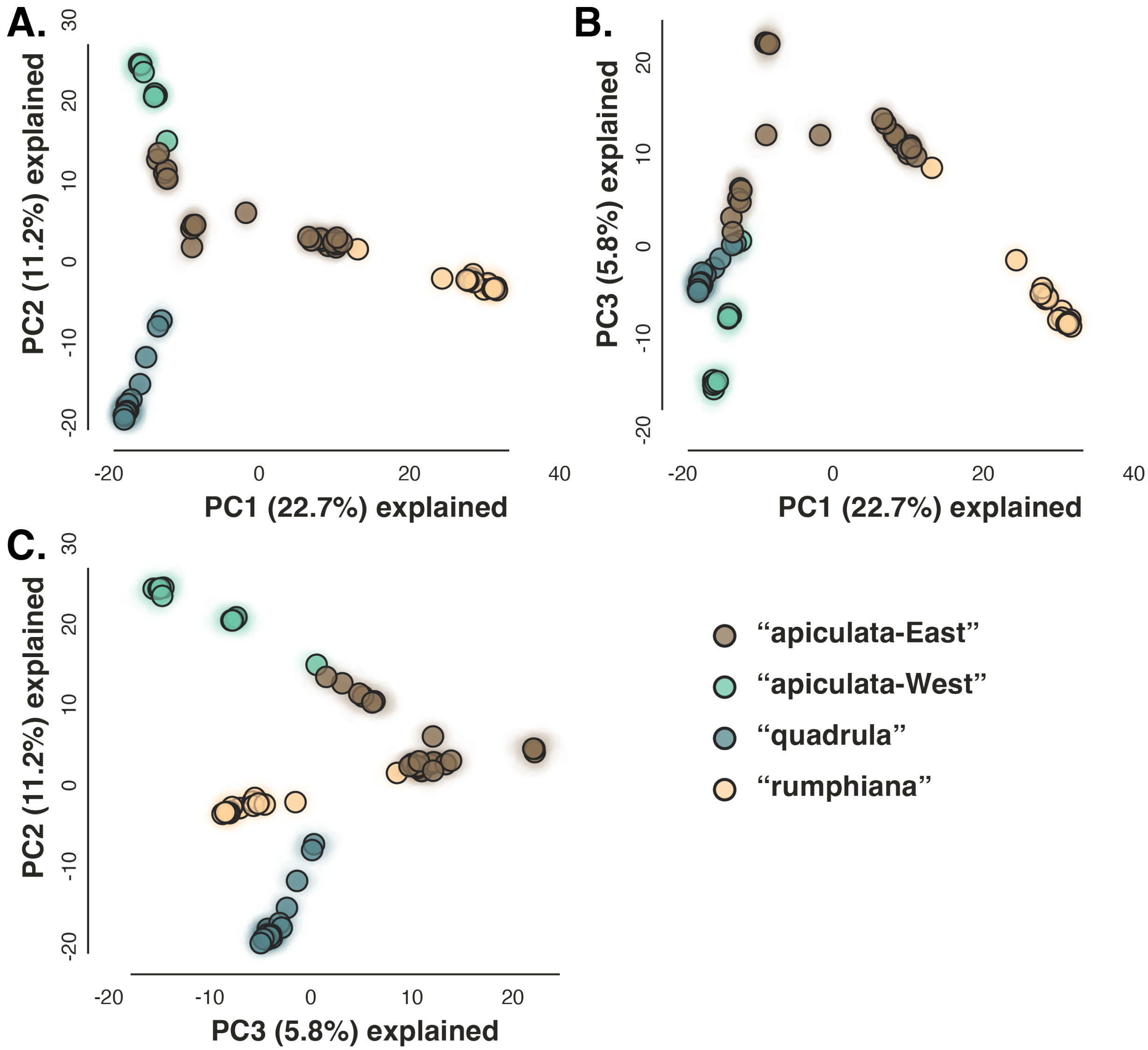

Supplementary Figure 8: Principal Component Analysis (PCA) plots of SNPs for all ingroup samples. Points are colored by majority sNMF ancestry. Smudge marks denote PC scores for each replication (N=100) of K-means clustering PCA.

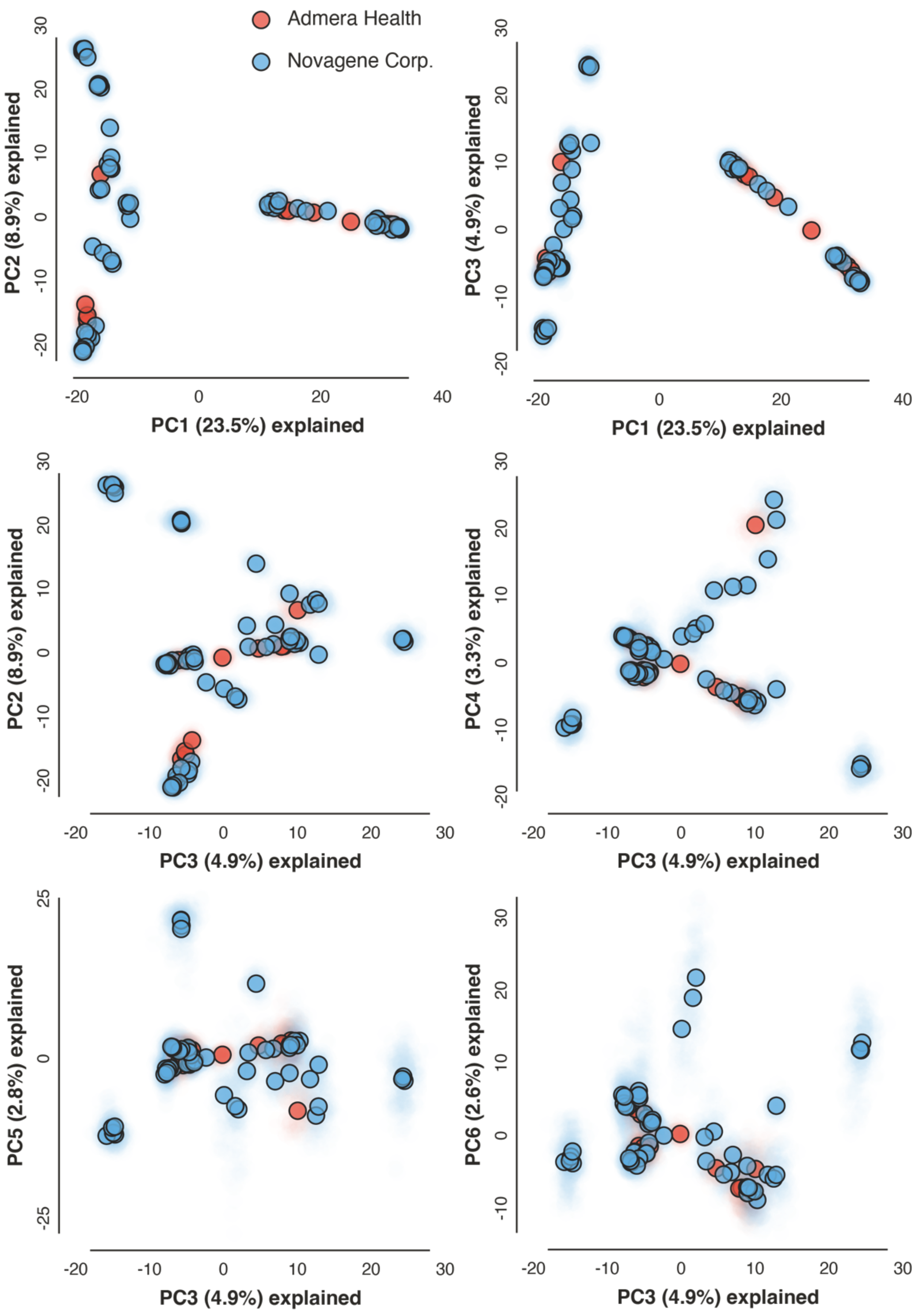

Supplementary Figure 9: Principal Component Analysis (PCA) plots of SNPs (nuDNA) colored by sequencing run.

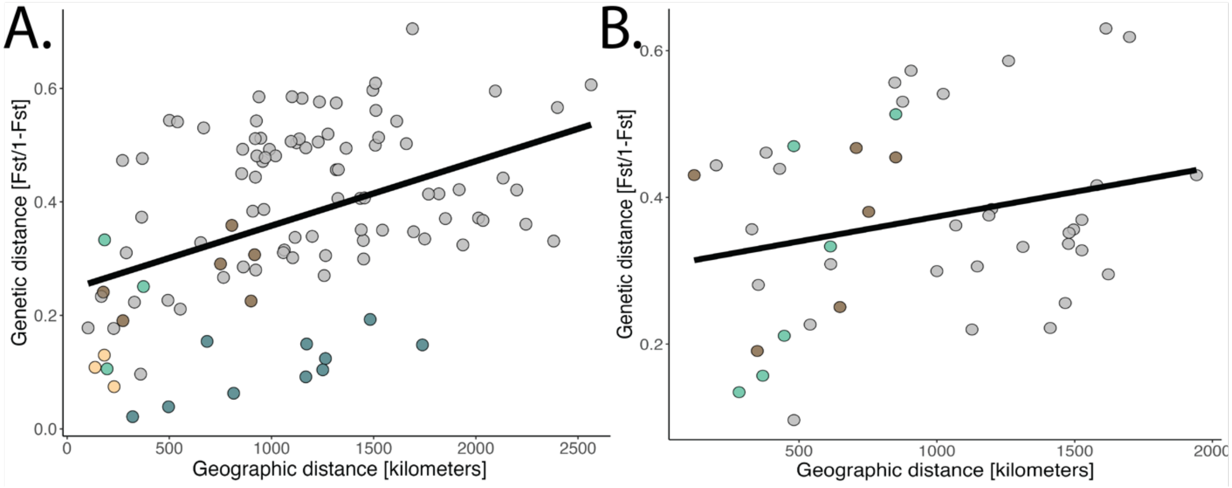

Supplementary Figure 10: Isolation by distance for all ingroup samples using SNPs (nuDNA) for (A.) Euclidean distance and (B.) clustered by drainage. Comparisons between different populations (i.e. "apiculata-East", "apiculata-West", "quadrula", and "rumphiana") are colored gray. Within population comparisons are colored consistently with Supplemental Figure 6 and 8. Black line is the global regression for all samples.

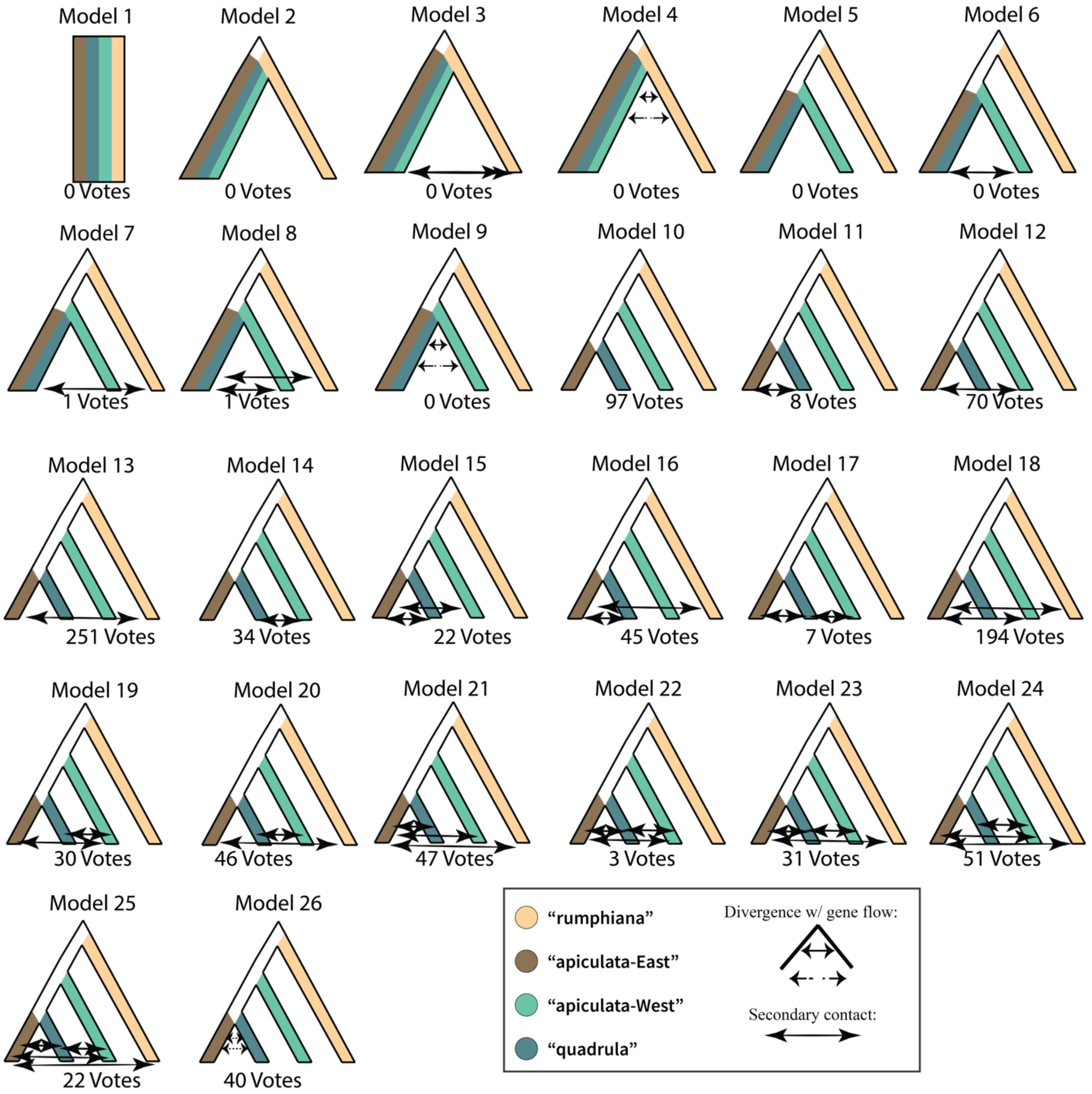

Supplementary Figure 11: All 26 speciation and demographic models tested by *delimitR* with the number of votes (model support) for each model. SNPs (nuDNA) were used to generate the input site frequency spectrum file.

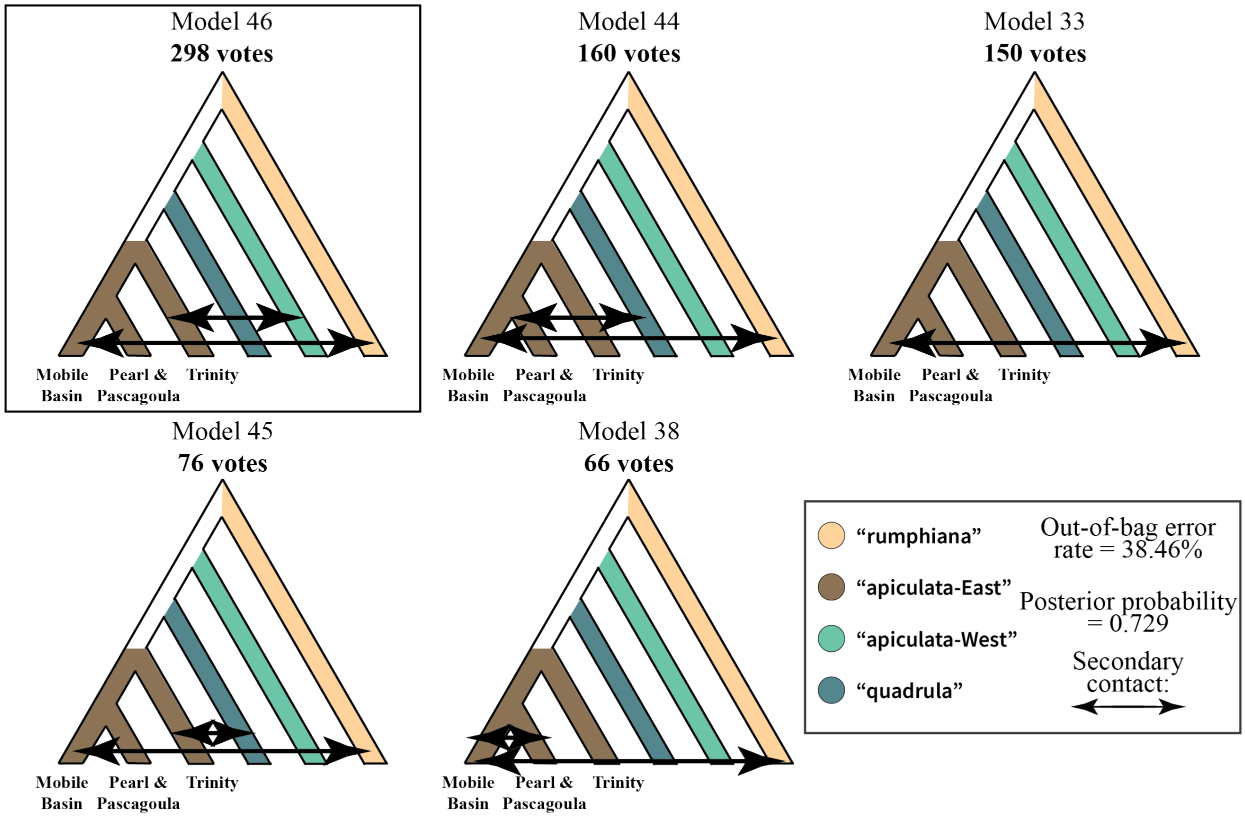

Supplementary Figure 12: Top five supported models by *delimitR* for the expanded ‘six-species’ analysis. SNPs (nuDNA) were used to generate the input site frequency spectrum file.
